# The Role of Noncoding RNAs in B-Cell Lymphoma

**DOI:** 10.3389/fonc.2020.577890

**Published:** 2020-10-23

**Authors:** Jingwen Li, Jing Zou, Xiaoyue Wan, Chunyan Sun, Fei Peng, Zhangbo Chu, Yu Hu

**Affiliations:** ^1^Institute of Hematology, Union Hospital, Tongji Medical College, Huazhong University of Science and Technology, Wuhan, China; ^2^Collaborative Innovation Center of Hematology, Huazhong University of Science and Technology, Wuhan, China

**Keywords:** B-cell lymphoma, microRNA, long-noncoding RNA, circular RNA, B cell development

## Abstract

In recent years, emerging evidence has suggested that noncoding RNAs (ncRNAs) participate in nearly every aspect of biological processes and play a crucial role in the genesis and progression of numerous tumors, including B-cell lymphoma. The exploration of ncRNA dysregulations and their functions in B-cell lymphoma provides new insights into lymphoma pathogenesis and is essential for indicating future clinical trials and optimizing the diagnostic and therapeutic strategies. In this review, we summarize the role of ncRNAs in B-cell lymphoma and discuss their potential in clinical applications.

## Introduction

B-cell lymphomas are a group of B-cell neoplasms with distinct natural histories and clinical behaviors. In general, B-cell lymphomas can be classified into B-cell non-Hodgkin lymphoma and Hodgkin lymphoma (HL) and are further categorized into dozens of subtypes based on their pathological, genetic, and clinical features. Despite novel effective therapies emerging in recent years, specific lymphoma subgroups remain incurable. Insights into the molecular mechanism of lymphoma onset and development will improve the understanding of their pathobiology and help develop personalized therapeutic strategies. During the past few decades, with the development of genetic and cytogenetic analysis technologies, accumulating evidence suggests that the large family of ncRNAs, which were once considered as “junk RNA,” play an essential role in the molecular events of B-cell lymphoma.

ncRNAs, which account for approximately 98% of the human transcriptome (1), are transcripts that do not encode proteins. Based on their length and structure, the majority of ncRNAs can be generally classified into several subtypes, including microRNA (miRNA), long noncoding RNA (lncRNA), and circular RNA (circRNA). Since the relevance between ncRNA and lymphoma pathogenesis first reported in 2002 (2), there have been increasing studies about the role of ncRNAs in lymphoma initiation and progression. In this review, firstly, we introduce the general characteristics of miRNAs, lncRNAs, and circRNAs and provide an overview of ncRNA functions in normal B-cell development. Then we discuss the current knowledge of deregulated ncRNAs in B-cell lymphoma, focusing on the most common lymphoma subtypes and mechanistically confirmed ncRNAs. Finally, we provide an overview of the clinical therapeutic potential of ncRNAs. Deciphering the critical role of ncRNAs in B-cell lymphoma may help improve the understanding of the molecular mechanism underlying the disease behavior and provide new perspectives for the diagnosis and treatment of B-cell lymphoma.

## Definition and Functions of ncRNAs

### miRNA

miRNAs are short RNA molecules consisting of 20–25 nucleotides. The processing of miRNAs is evolutionally conserved. Briefly, after transcribed by RNA polymerase II (RNA Pol II) (3), the initial transcripts, which are known as primary miRNAs, undergo a multi-step process in which their length is reduced to ~22 nt by RNA endonucleases including Drosha and Dicer. The processed mature miRNAs are assembled into the RNA-induced silencing complex (RISC) by interacting with Argonaute proteins and exert the posttranscriptional regulatory functions through binding to the complementary regions located in the 3’-untranslated region of their target mRNAs and inducing silencing and degradation of these transcripts (4). miRNAs mainly act as inhibitors of gene expression, though under some circumstances, they may also function as activators. To note that, miRNA expression can be regulated by transcriptional modifiers, which can also be miRNA targets. Accumulating evidence has suggested that some miRNAs and their target molecules form autoregulatory feedback loops, which are involved in tumor biology. It was reported that more than 60% of human genes are under the regulation of miRNAs, and as a consequence, miRNAs participate in most cellular events.

### lncRNA

lncRNAs are noncoding transcripts of more than 200 nucleotides in length. According to their genomic location, lncRNAs can be divided into several types, including intergenic lncRNAs, enhancer lncRNAs, and lncRNAs intersecting other genes on the sense or antisense strand (antisense lncRNAs) (5). lncRNAs have many similarities with mRNAs regarding their processing, since most lncRNAs are transcribed by RNA Pol II, capped in the 5’ end, and are polyadenylated in the 3ʹ end (5). In addition, lncRNAs are characterized by the secondary and tertiary structure as a result of Watson–Crick base pairing. The binding ability with the complementary sequence and the spatial conformation provide lncRNAs with biological functions as nucleic acid as well as protein. lncRNA expression represents a tissue- and time-specific manner and may be altered in response to diverse stimuli. Thus they may act as essential mediators of signal transduction. lncRNAs could interact with DNA, RNA, and protein and regulate the expression and function of these molecules through various mechanisms. The binding of lncRNAs to DNA in protein-coding or noncoding regions, including regulatory sequences such as promoters and enhancers, is often observed in lncRNAs located in the nucleus, which allows lncRNAs controlling gene expression *via* cis- or trans-regulation. For example, lncRNA TUG1 directly binds to the promoter of the PGC-1α gene and induces PGC-1α transcription (6). The regulation of lncRNAs on gene transcription through interacting with DNA is usually dependent on the lncRNA-DNA hybrid, which alters the structure of chromatin and influences the recruitment of transcriptional modifiers (7). Besides, the binding of lncRNAs to DNA may lead to concomitant transcription of both lncRNA and mRNA, and consequent collision or stalling of RNA Pol II, thus transcription repression (8). The interaction between lncRNA and mRNA is associated with intron retention and alternative RNA splicing (9). lncRNAs with different domains, which allow the concomitant combination of various proteins, act as scaffolds to assist the assembling of multi-protein complexes, such as chromatin remodeling complexes, and guide the interaction between protein and DNA or RNA. It was shown that upon inflammatory stimulation, lncRNA FIRRE is upregulated and stabilizes mRNAs of the target inflammatory genes through recruiting hnRNP U protein (10). lncRNAs also participate in epigenetic modifications through recruiting modifiers to certain genes. For example, lncRNA MALAT1 has been reported to interact with the enhancer of zeste homolog 2 (EZH2) and induce H3K27me3 modification of its target genes in various tumors (11). Furthermore, lncRNAs can act as decoys that negatively regulate the functions of the effector molecules. The binding of lncRNAs may impact the conformation, stability, and localization of their targets. Through the numerous regulatory mechanisms, lncRNAs play a crucial role in various biological processes, including cell proliferation, differentiation, DNA repairing, apoptosis, and autophagy. The dysregulation of lncRNAs has been correlated with diverse human disorders (12). Nowadays, more than 50,000 lncRNAs have been recognized, and the list of identified lncRNA loci as well as lncRNA isoforms is continuing to expand.

### circRNA

circRNAs, a group of highly conserved ncRNAs, have been increasingly gaining attention from cancer research to biotechnology during recent years. Different from linear RNAs, circRNAs have closed circular structure with a phosphodiester bond between the 5’- and 3’-end of the transcript, which is formed through a back-splicing reaction (13). Lack of free ends provides circRNAs with high stability against exonucleases. Similar to lncRNA, the expression of circRNA represents a tissue- and time-specific manner. Studies using next-generation sequencing showed specific expression patterns of circRNAs in human cancers (12), suggesting that they may play a role in tumor pathogenesis. Diverse cellular functions of circRNAs have been validated. circRNAs with certain miRNA-binding sites can indirectly regulate gene expression through sponging their complementary miRNAs (14). In addition, circRNAs may interact with proteins, act as protein decoys or scaffolds, and perform other functions such as sequestering or storing their binding proteins (15). Moreover, some circRNAs are suggested to participate in tumorigenesis through encoding regulatory peptides (16), yet the majority of circRNAs are considered as noncoding RNAs. Though the understanding of their functions is still at the primary stage, there is no doubt that circRNAs are important players in regulating cellular biology and have the potential to participate in every aspect of oncogenic processes.

## The Interaction Between Different ncRNAs

The interaction between different ncRNAs through complementary base-pairing represents a critical mechanism underlying cellular events. The direct binding of lncRNA or circRNA to miRNA prevents the interaction of miRNA with their target mRNAs, which is known as the mechanism of competing endogenous RNA (ceRNA). One lncRNA or circRNA may sponge various miRNAs *via* different binding sites. For example, lncRNA MALAT1 has been reported to target and repress miR-150 and miR-101 (17, 18). Inhibition of lncRNA MALAT1 releases its suppressive effect on these miRNAs, thus activation of mRNAs targeted by miR-150 and miR-101. Additionally, the interaction between miRNA and lncRNA may impact the function of the lncRNA. It was shown that specific mutations in LINC00673 allowed the binding of miR-1231, which suppressed the antitumor function of this lncRNA (19).

## ncRNAs in B-Cell Development

In recent years, studies using genetically modified mice have highlighted the role of ncRNAs in B-cell development. B-cell development can be divided into a series of sequential and highly regulated processes. Early B-cell development in the bone marrow (BM) includes pro- and pre-B cell stages. During these stages, B cells undergo rearrangement of immunoglobulin (Ig) heavy (H) chain and light (L) chain genes and differentiate into mature naïve B cells that express functional B-cell receptors (BCRs) without auto-immune activity. Then mature naïve B cells leave from the BM to the secondary lymph tissues, where they are exposed to antigens. Some activated B cells differentiate into IgM-secreting B cells. Others enter the follicles and form the germinal centers (GCs), where they undergo a series of ordered events, including somatic hypermutations and class switch recombination, and finally become plasma cells and memory cells. These processes are tightly controlled by a complex network of transcription factors, some of which were reported to participate in tumorigenesis. Similarly, some ncRNAs identified as important players during normal B-cell development may also be dysregulated and participate in pathological processes of lymphoma. Exploration of ncRNA expression and function in normal B-cell development may help improve the understanding of their pathogenetic role in B-cell lymphoma.

Several miRNAs represent key regulators in B-cell lineage differentiation. For example, miR-181a is the first miRNA reported to play a role in regulating B-lineage differentiation. Enforced miR-181a expression in the hematopoietic stem cells or progenitor cells upregulated the fraction of B-lineage cells both *in vitro* and *in vivo* (20). During early B-cell development, the miR-17-92 cluster, which contains six miRNAs processed from the same precursor transcript (miR-17, miR-18a, miR-19a/b-1, miR-20a, and miR-92-1), and two paralogs (the miR-106b-25 cluster and the miR-106a-363 cluster), promotes B-cell transition from pro- to pre-B cell, probably through targeting pro-apoptotic protein PTEN and Bim (21). Deficiency in miR-17-92 cluster inhibits pro-B cells developing into pre-B cells (21). In addition, several miRNAs negatively regulate the transition from pro- to pre-B stage. miR-34a is most highly expressed at the pro-B stage. Ectopic expression of miR-34a is related to decreased formation of mature B cells, which is mediated by its direct target forkhead box P1 (Foxp1), a transcription factor required for B-cell differentiation (22). Similarly, miR-150 was reported to target Foxp1 (23), though mainly in mature B cells. It was shown that miR-150 targeted c-Myb in pro-B cells (24–26). Enforced expression of miR-150 in hematopoietic progenitors impairs mature B-cell formation (26). miR-212/132 cluster negatively regulates pro-B cell survival through targeting Sry−Related HMG−BOX−4 (Sox4), a transcription factor necessary for pro-B cell survival (27). A more recent study showed that miR-125b, which is expressed in BM multipotent progenitors and myeloid cells, targeted sphingosine-1-phosphate receptor 1 (S1PR1), a key regulator during the release of immature B cells from BM (28). GC B-cell development is also modulated by several miRNAs. miR-155 is a critical controller miRNA in GC reactions. miR-155 deletion leads to impaired GC-B cell formation and IgG1 switching (29, 30). Mechanistically, miR-155 targets the hematopoietic transcription factor PU.1, and thus regulates the maturation of IgG1 cells (29). Studies also showed that miRNA-155 fine-tunes the expression of activation-induced cytidine deaminase (AID), which acts as a key player in somatic hypermutation and Ig class switching (31). In addition, AID is targeted by miR-181b (32), another chief controller miRNA in GC B cells (25). Overexpression of miR-181b in premature B cells impairs B-cell class switch recombination through downregulating AID. Moreover, miR-28 negatively regulates the survival of GC cells *via* modulating a series of genes associated with BCR signaling and cell proliferation (33). miR-217 positively regulates GC reaction *via* reducing DNA damage responses and stabilizing Bcl-6 expression (34).

**Figure 1:**
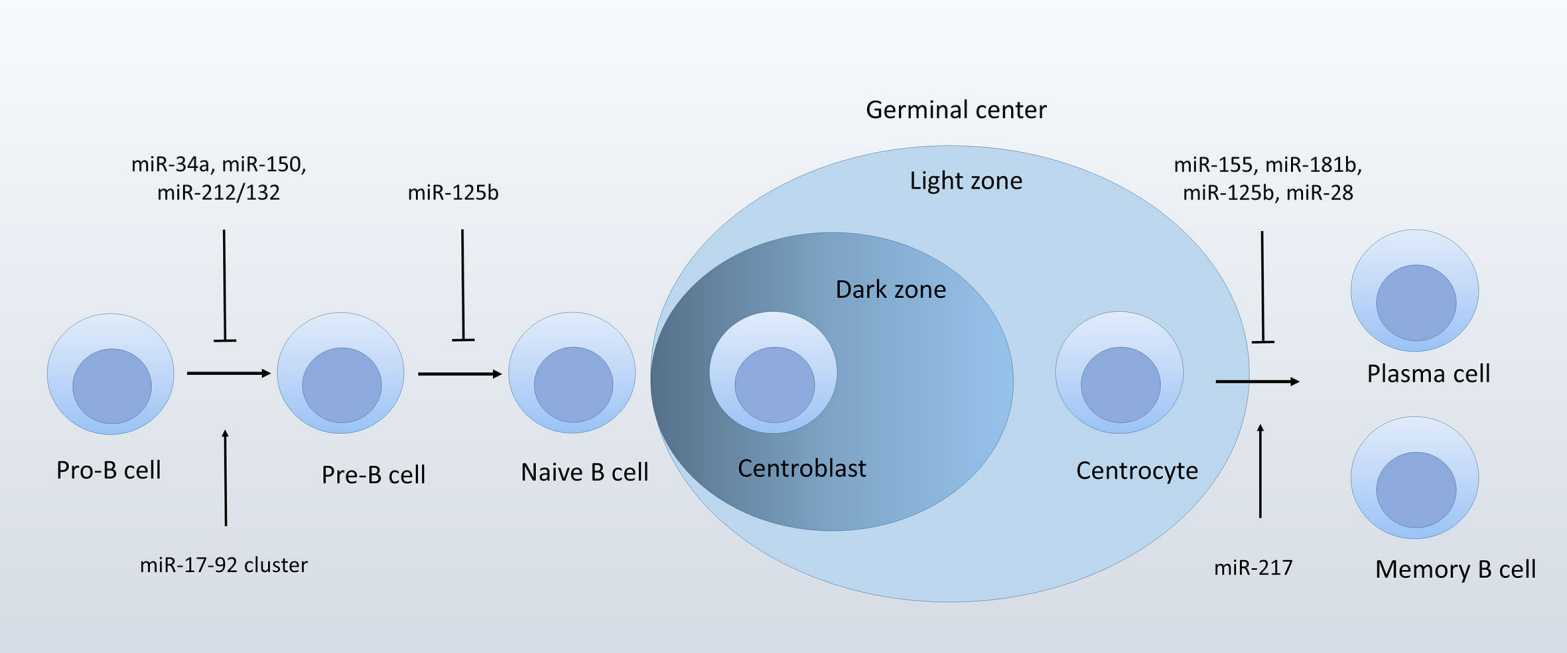
ncRNAs regulate normal B-cell development.

There is still little known about the role of individual lncRNA or circRNA in B-cell development. A study reported the high expression of lncRNA colorectal neoplasia differentially expressed (CRNDE) in proliferating GC B cells (35). Though the biological function of CRNDE in B-cell development has not been examined, studies showed that CRNDE regulates the central metabolism and promotes metabolic changes in proliferative cells (36). The finding is consistent with its high expression in centroblasts of GCs, suggesting that CRNDE may play a role as a metabolic regulator during B-cell development. The noncoding antisense transcripts of *PU.1* were reported to inhibit PU.1 at a level of translation (37). Considering the crucial role of PU.1 in B-cell differentiation (38), it can be assumed that the antisense PU.1 may regulate B-cell development through modulating PU.1 expression (**Figure 1**).

ncRNAs participate in nearly every stage of B-cell development. Thus, it’s not surprising that ncRNA dysregulations contribute to lymphomagenesis and development. The role of ncRNAs in B-cell lymphomas has been elucidated by multiple studies (39), mainly from two experimental approaches: ncRNA expression pattern in specific B-cell lymphoma, and functions and underlying mechanisms of dysregulated ncRNAs in lymphoma cell lines or murine models. Below we will discuss the pathogenic role of ncRNAs in B-cell lymphoma mainly from these two aspects.

## ncRNA Dysregulations in B-Cell Lymphoma

In recent years, studies using high-throughput technologies and the polymerase chain reaction explored ncRNA expression in B-cell lymphoma and identified certain ncRNAs that are frequently deregulated. Some dysregulated ncRNAs (compared with the normal counterpart) with validated functions in common types of B-cell lymphoma are listed in **Table 1**.

**Table 1:** Some dysregulated ncRNAs with validated functions in the common subtypes of B-cell lymphoma.

Lymphoma subtype	ncRNA	ncRNA class	Expression	References
DLBCL	miR-15a/16, miR-27b, miR-28, miR-34a, miR-101, miR-124, miR-150, miR-181a	miRNA	↓	(40–45),
	miR-17-92 cluster, miR-21, miR-155, miR-146a/b, miR-217		↑	(34, 40–42),
	SMAD5-AS1, PANDA, FAS-AS1, lincRNA-21, TUG1	lncRNA	↓	(46–50),
	SNHG14, OR3A4, NEAT1, FIRRE, MALAT1, HOTAIR, LUNAR1, SMAD5-AS1, HULC		↑	(48, 51–57),
	circ-APC	circRNA	↓	(58)
CLL	miR-15a/16-1, miR-26a, miR-28, miR-29, miR-30a, miR-34a, miR-125b, miR-150, miR-181a/b, let-7 family	miRNA	↓	(2, 23, 42, 59–63),
	miR-17-92 cluster, miR-21, miR-22, miR-155, miR-221/222		↑	(59, 61, 64),
	CANDE, FAS-AS1	lncRNA	↓	(49, 65),
	circ-CBFB	circRNA	↑	(66)
BL	miR-15/16, miR-26, miR-28, miR-29, miR-150	miRNA	↓	(67–70),
	miR-17-92 cluster, miR-21, miR-194, miR-217		↑	(34, 67, 70, 71),
	FAS-AS1	lncRNA	↓	(49)
	MINCR		↑	(72)
	ZDHHC11, ZDHH11B	circRNA	↑	(73)
FL	miR-26a, miR-28, miR-29c, miR-149, miR-150, miR-181a	miRNA	↓	(41, 74–76),
	miR-9, miR-20a/b, miR-106a, miR-155, miR-194, miR-494		↑	(41, 70, 77–79),
MCL	miR-26a, miR-29, miR-150	miRNA	↓	(63, 80),
	miR-17-92 cluster, miR-146a, miR-222		↑	(81–83),
	SNHG12	lncRNA	↓	(84)
	HAGLROS, MANCR, ROR1-AS1, MALAT1		↑	(11, 85–87),
	CircCDYL	circRNA	↑	(88)

In diffuse large B-cell lymphoma (DLBCL), the most common invasive NHL, more than 100 miRNAs have been reported to be deregulated (40, 41, 89, 90), including numerous miRNAs with identified biological functions, such as miR-15a/16, miR-21, miR-34a, miR-106, miR-146a/b, miR-150, and miR-155 (91–98). A study reported 2,632 novel lncRNAs specifically expressed in DLBCL clinical samples compared with normal B cells, nearly half of which were differentially expressed and clustered across different groups of DLBCL samples (99), suggesting that these lncRNAs may participate in DLBCL pathogenesis. Single lncRNA with known biological functions has been studied in DLBCL. For example, overexpression of MALAT1, one of the most widely studied lncRNAs, was confirmed in DLBCL.

Based on the genomic characteristics, DLBCL can be generally divided into two subgroups, germinal center B‐cell‐like DLBCL (GCB‐DLBCL) and activated B‐cell‐like DLBCL (ABC‐DLBCL) (100). These two subgroups are associated with different clinical outcomes. A previous study reported a miRNA signature, including miR-155, miR-21, and miR-221, to distinguish GCB‐ and non‐GCB DLBCL (90). In particular, a higher expression of miR-155 in ABC‐DLBCL was confirmed by several independent studies (41, 64, 90, 101, 102). A recent study using the data from the GEO database revealed 156 lncRNAs differentially expressed between GCB- and ABC-DLBCL and identified 17 lncRNA to distinguish these two subgroups (46).

Burkitt lymphoma (BL) is characterized by high aggressiveness and fast-growing ability. Abnormal expression of c-Myc is a frequent event in BL due to the translocation of *c-Myc* and immunoglobulin genes. It’s not surprising that miRNAs controlled by c-Myc, such as miR-23a/b, miR-34b, miR-125b, miR-17-92 cluster, and let-7 family are deregulated in BL (103–105). A recent study identified a group of c-Myc-induced lncRNAs, which were differentially expressed in BL patient samples compared with normal GC B cells (72). The distinction between DLBCL and BL is clinically significant due to their different treatment protocols and clinical outcomes. However, these two subtypes cannot be reliably distinguished from each other based on the WHO definition, and specific genomic profiling is established to fulfill the diagnostic criteria (106). Studies have revealed specific miRNA signatures to distinguish BL from DLBCL (42, 107). Notably, many of these miRNAs are correlated with Myc or NF-kB signaling.

Studies of miRNAs in chronic lymphocytic leukemia (CLL) largely improved our understanding of the role of miRNAs in tumor pathogenesis, since Calin et al. first reported that miR-15a/16-1 cluster is located in 13q14 region (2), which is frequently deleted in CLL patients (108). The further functional analysis confirmed the association between miR-15a/16-1 downregulation and CLL pathogenesis (109, 110). Other ncRNAs with validated biological functions, such as miR-21, miR-34a, miR-150, miR-155 (59, 111–119), and MIAT, were also reported to be deregulated in CLL (120).

Several studies explored miRNA expression patterns in follicular lymphoma (FL) (77). A study reported 17 miRNAs differentially expressed between t(14;18) (q32;q21)-positive and -negative FL patients and revealed that these miRNAs were associated with the expression of multiple genes involved in cell proliferation, differentiation, and apoptosis (74). A study explored lncRNA expression in FL and identified 189 significantly deregulated lncRNAs. Among these lncRNAs, ENST00000545410 (RP11-625 L16.3) was confirmed to play a pathogenetic role (121).

So far, there are few studies about circRNA expression profiling in B-cell lymphomas. A recent study using RNA-seq and NanoString assay provided a landscape of circRNA expression in mantle cell lymphoma (MCL) (122).

## Mechanisms of ncRNA Dysregulations in B-Cell Lymphoma

### Genetic Alterations

Genetic alterations, which are frequently acquired by B-cell lymphoma, have been correlated with ncRNA dysregulations. Studies using comparative genomic hybridization reported an association between global miRNA expression profiling and DNA gain or loss in B-cell lymphomas (123, 124). However, it seemed that alterations in copy number alone are not able to explain the majority of miRNA expression changes. Downregulation of miR-15a/16-1 is associated with 13q14 deletion, which is often observed in solid and hematological malignancies, particularly in CLL. Highly expressed miR-17-92 cluster in DLBCL, MCL, and BL is correlated with amplification in the MIHG1 locus cluster on 13q31 (81, 125, 126). Studies exploring the association between chromosomal translocation and ncRNA deregulation showed that transcriptional activation of miR-125b-1 (located in chromosome 11) was correlated with t(11,14)(q24;32) translocation (127). Finally, DNA mutations contribute to ncRNA dysregulations. A study identified germline or somatic mutations in several miRNAs, including miR-16-1, miR-27b, miR-29b-2, miR-187, and miR-206 in CLL (119). Further analysis showed that germline mutations in the primary precursor of miR-15a/16-1 led to significantly decreased expression of miR-15a/16-1. Another study by Kminkova et al. reported that in CLL patients, specific mutations and SNPs presented in miRNA genes, including miR-16-1 and miR-29b/c, might alter the expression of these miRNAs (128). Most of the variations were found located outside the region of mature miRNAs and change the secondary structures of pri- or pre-miRNAs. Those changes may influence miRNA processing machinery, thus regulating their expression (128). In addition, previous studies reported mutations in miR-142 in DLBCL. Some mutations in the seed sequence of miR-142 generated novel potential binding sites of ZEB2, a transcriptional repressor associated with EBV proliferation, and some impaired the combination of miR-142 to its targets, including RAC1 and ADCY (129).

### Epigenetic Alterations

Epigenetic dysregulations, which are controlled by a number of complexes, including DNA methyltransferases and histone modifiers, may lead to abnormal ncRNA expression. Promoter hypermethylation may suppress ncRNA expression. For example, downregulation of miR-139 and miR-582 in CLL (130), of miR-29 in BL, and of miR-124-1 and miR-203 in various B-cell lymphomas are correlated with hypermethylation at their promoters (69, 131, 132). Promoter hypermethylation may also induce dysregulations of lncRNA, the lower expression of lncRNA CANDE in CLL, for example (65).

Histone modifications, including di- or trimethylation, and acetylation are associated with abnormalities in ncRNA transcription. For example, overexpression of EZH2 induces H3K27me3 at the promoter of lncRNA FAS-AS1, thus FAS-AS1 downregulation (49). Besides, histone deacetylases (HDACs) trigger histone deacetylation and consequent gene silencing. Overexpression of HDACs downregulates miR-15a/16-1 and miR-29b in a group of CLL patients (133). Several studies confirmed that miR-15a/16-1 and miR-29a/b/c were epigenetically repressed by HDAC3 and EZH2, under the facilitation of c-Myc, in several lymphoma cell lines (134, 135).

### Transcriptional Abnormalities

Enhanced or suppressed transcription by conventional transcription factors contributes to ncRNA dysregulations. c-Myc is frequently deregulated in B-cell lymphomas, particularly in BL. Many miRNAs have been reported to be regulated by c-Myc at a level of transcription, the miR-17-92 cluster, for example (136). Besides, miR-150 is downregulated by c-Myc in FL, which is associated with FL transformation (75). Other miRNAs involved in B-cell lymphoma pathogenesis such as miR-29, miR-34, miR-125b, miR-146a, miR-155, and let-7a are also targeted by c-Myc (137–139). Interestingly, it was shown that c-Myc induced the overexpression of circRNAs ZDHHC11 and ZDHHC11B, which sponge and suppress miR-150 in BL cells. The finding suggested that c-Myc downregulated miR-150 *via* at least two mechanisms to ensure low expression of miR-150 and subsequent elevated level of its target c-Myb (73). Additionally, a study performing RNA-seq in BL samples and cell lines revealed 13 lncRNAs regulated by c-Myc. Among them, the level of one lncRNA located in chromosomal 8 was significantly associated with c-Myc expression, due to which it was named Myc-induced long noncoding RNA (MINCR) (72). Several lncRNAs, including NEAT1 and FIRRE, were reported to be induced by c-Myc in DLBCL (53, 54).

In addition to c-Myc, p53 abnormalities are also correlated with the deregulation of numerous ncRNAs in B-cell lymphomas. miR-34, which is identified as a key p53 target in various tumor types, including lymphomas, represents a lower expression in CLL with p53 inactivation (60, 140). miR-34a was upregulated after induction of DNA damage response and p53 stabilization in CLL (60, 141). Downregulation of miR-34 increased cell viability after DNA damage, thus related to the resistance of lymphoma cells to chemotherapy. miR-15a/16-1 cluster and miR-124 were reported to be directly targeted and activated by p53 (142, 143). Enforced expression of miR-15a/16-1 downregulates p53 and consequently impacts miR-34a/b/c expression in lymphoma cells (142), thus forming a miRNA/p53 feedback circuity. lncRNA NEAT1 and lincRNA-p21 were reported to be upregulated upon p53 induction (144). Decreased expression of lncRNA PANDA is related to p53 downregulation in DLBCL (46). Also, upregulated c-Myb contributes to the overexpression of miR-155 by stimulating its transcription (114). miR-155 is also a transcriptional target of NF-κB (68). Epstein-Barr virus (EBV)-encoded latent membrane protein-1 (LMP1) upregulates miR-155 through enhancing NF-κB activation (145). Studies also showed NF-κB knockdown reduces miR-21 level in B-cell lymphoma, suggesting that deregulated NF-κB may lead to the increased expression of miR-21 (146).

### Processing Abnormalities

As we have mentioned, miRNAs are processed from long primary precursors known as pri-miRNAs. Aberrant miRNA processing may lead to reduced expression of tumor-suppressive miRNAs in B-cell lymphomas. It was observed that the primary transcript of miR-15a/16-1 was upregulated, whereas the precursor was downregulated in CLL (147), suggesting a block in DROSHA-induced cleavage of miR-15a/16-1. Further analysis confirmed that the defect in pri-miRNA processing was induced by RNA-specific deaminase ADARB1, probably through interacting DROSHA and competing with its RNA-processing activity (147). Besides, processing abnormalities seem to contribute to miR-155 downregulation in BL. Forced introduction of pri-miR-155 in BL cell lines does not increase miR-155 expression, while it upregulates miR-155 in normal B and HL cells (68), suggesting that the expression of mature miR-155 is downregulated during its processing in BL cells.

### Virus-Induced ncRNA Dysregulation

**Figure 2:**
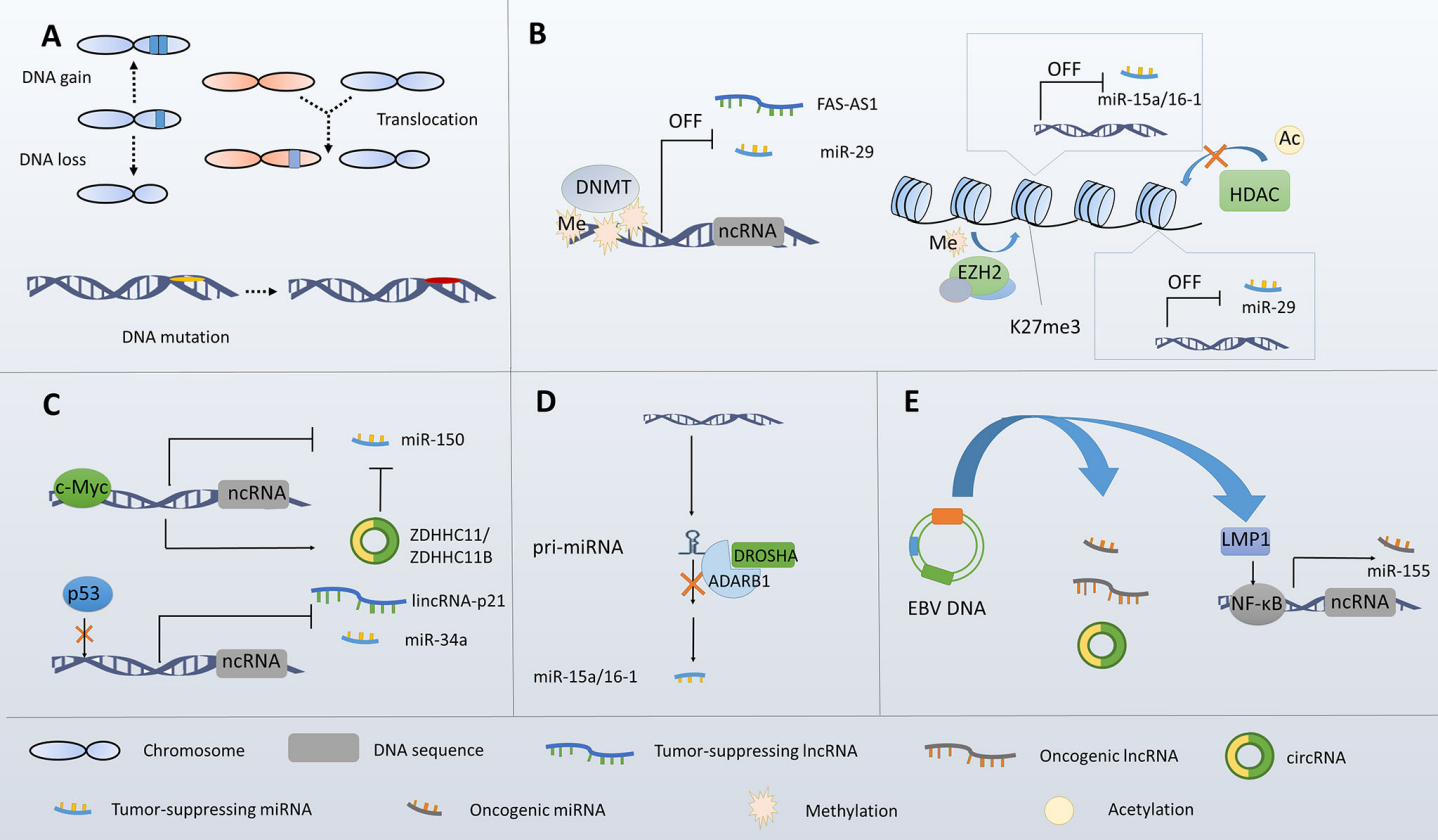
Overview of the mechanisms underlying ncRNA dysregulation in B-cell lymphoma. **(A)** Genetic alterations lead to ncRNA dysregulations. **(B)** Epigenetic alterations lead to ncRNA dysregulations. **(C)** Transcriptional abnormalities lead to ncRNA dysregulations. **(D)** Processing abnormalities lead to ncRNA dysregulations. **(E)** EBV infection leads to ncRNA dysregulations.

The infection of EBV, a DNA virus of the herpesvirus family, has been correlated with the genesis of several lymphomas, including BL, HL, and DLBCL. EBV infection may influence cellular ncRNA expression. The initial evidence came from the findings that the miRNA signature differs between EBV-positive and -negative BL (68). Specific EBV-encoded proteins have been confirmed to participate in ncRNA regulations. As previously mentioned, miR-155 is induced by LMP1-mediated NF-κB activation. Similarly, miR-146 and miR-29b are upregulated by LMP1 in EBV-positive lymphomas (148, 149). Besides, miR-146a, miR-21, miR-34a, and miR-155 were reported to be deregulated by Epstein-Barr nuclear antigen 2 (EBNA2) (150–152). Interestingly, a recent study using Ago-IP and RNA sequencing suggested EBV infection of DLBCL cells not only influencing miRNA expression but also the overall content of miRNA in Ago2 complex, a complex through which miRNAs are tethered to their target mRNAs (153). It’s worth noting that EBV encoded ncRNAs, including miRNAs and lncRNAs, may participate in lymphoma onset and development, which has been demonstrated by several studies (154–158). In addition, a recent study identified an EBV-encoded circRNA named ebv_circ_RPMS1, which was suggested to sponge miRNAs with validated biologic or pathogenetic functions (159) (**Figure 2**).

Kaposi’s sarcoma-associated herpesvirus (KSHV/HHV8) is strongly correlated with the development of a specific lymphoma subset, the primary effusion lymphoma (PEL). KSHV encoded ncRNAs may act as an important mediator in KSHV-induced lymphomagenesis. Some KSHV-encoded miRNAs were reported to mimic the function of cellular miRNAs, including miR-155 and miR-142, and participate in the regulation of various signaling pathways (160). A recent study revealed that numerous circRNAs were encoded by the lytic genes of KSHV (161). Functions of these circRNAs in PEL pathogenesis need to be further explored in the future.

## Functions and Underlying Molecular Mechanisms of ncRNAs in B-Lymphoma

### ncRNAs Regulate Cell Apoptosis in B-Cell Lymphoma

#### ncRNAs Regulate Cell Apoptosis Through Modulating Bcl-2 Family

The B-cell lymphoma-2 (Bcl-2) family, which plays a key role in modulating cell apoptosis *via* the mitochondrial death machinery, consists of both anti-apoptotic or pro-apoptotic proteins, and the balance between these proteinsare is strictly regulated by ncRNAs. Studies showed that miR-15a/16-1 regulated cell apoptosis *via* directly targeting and downregulating Bcl-2 (109). Enforced expression of miR-15a/16-1 increased apoptosis of malignant B cells both *in vitro* and *in vivo*. In addition, lncRNA HULC was suggested to regulate Bcl-2 expression in B-cell lymphoma (55). Studies showed that HULC significantly overexpressed in DLBCL samples and cell lines. Knockdown of HULC induced apoptosis of DLBCL cells, which was accompanied by a decreased level of Bcl-2 (55). Myeloid cell leukemia-1 (Mcl-1) is a potent anti-apoptotic protein belonging to the Bcl-2 family. It was reported that the Mcl-1 transcript was targeted by miR-29b in several malignant B-cell lines. Ectopic expression of miR-29b reduces Mcl-1 expression, and consequently increases apoptosis of malignant B cells. Besides, Mcl-1 is one of the targets of miR-15a/16-1 (133). The decreased level of miR-15a/16-1 in p53-knockout mice, which developed aggressive CLL, was associated with Mcl-1 overexpression (162). miR-181a/b, significantly downregulated in CLL, targets both Mcl-1 and Bcl-2, thus modulating cell apoptosis (118). Besides, the lower expression of miR-181b is correlated with poorer prognosis of CLL patients (111, 118, 163). In addition, Bcl-xL is another anti-apoptotic protein belonging to the Bcl-2 family, and miR-135b was reported to downregulate Bcl-xL through targeting JAK2, a Bcl-xL activator (164). Moreover, the miR-17-92 cluster was confirmed to repress pro-apoptotic protein Bim, which contributed to its pathogenetic role in B-cell lymphoma (81).

#### ncRNAs Regulate Cell Apoptosis Through Modulating p53 Protein

P53 mediates diverse tumor-suppressive effects through modulating transcription of multiple genes involved in cell apoptosis, proliferation, and DNA repairing. Defective p53 signaling, which enables malignant cells to escape from apoptosis and cell cycle arrest, is strongly correlated with tumorigenesis and development across human malignancies. miR-34a is identified as a key regulator of p53 (60). Mechanistically, miR-34a targets SIRT1, which inhibits cell apoptosis through deactivating p53 (138, 165). Downregulation of miR-34a releases its suppressive effect on SIRT1, consequently leading to p53 inhibition. Since miR-34a transcription is activated by p53 (166), miR-34a, SIRT1, and p53 form a feedback loop that positively regulates p53 expression. In addition, a negative feedback loop is formed between the miR-15a/16-1 cluster and p53, since miR-15a/16-1 is transcriptionally activated by p53, and in turn it targets p53 and represses its expression (142). Additionally, p73, a member of p53 families, works synergistically with p53 to regulate cell apoptosis. Studies reported that miR-106b targeted ubiquitin ligase Itch. Upregulation of miR-106b by chemotherapy induced p73 accumulation and cell apoptosis in CLL (167).

#### ncRNAs Regulate Key Signaling Pathways Associated With Cell Survival

PI3K/Akt signaling provides potent anti-apoptotic effects in cells. Aberrant activation of PI3K/Akt signaling, which is frequently observed in B-cell lymphoma, is correlated with ncRNA dysregulations. It was reported that miR-17-92 cluster directly targeted and downregulated PH domain and leucine-rich repeat Protein Phosphatase 2 (PHLPP2) (81, 168), a negative regulator of PI3K/Akt signaling pathway. Overexpression of miR-17-92 was shown to activate PI3K/Akt signaling (81). Besides, miR-19a/b was also demonstrated to target PTEN, which negatively regulates Akt activation. Other miRNAs, including miR-21 and miR-22, were also reported to target PTEN in B-cell lymphomas (146, 169, 170). In addition, miR-27b, which is epigenetically silenced in DLBCL, targets tyrosine kinase MET and consequently repressed PI3K/Akt signaling. Forced introduction of miR-27b in DLBCL cells inhibits cell survival and proliferation (98). miR-155 was reported to activate PI3K/AKT signaling pathway in DLBCL and CLL through targeting SH2-containing inositol phosphatase-1 (SHIP-1) (171, 172). Increased miR-155 expression and consequent Akt activation are associated with chemoresistance of DLBCL cells (42, 173). Furthermore, miR-34a was shown to target Axl, a tyrosine receptor kinase activating PI3K/Akt signaling and overexpressed in CLL (174). Moreover, T-cell leukemia/lymphoma 1 (Tcl1) is a coactivator of Akt (175). High expression of Tcl1 was found in CLL, BL, and DLBCL, particularly in aggressive CLL subtypes (116). Several miRNAs, including miR-29b, miR-181b, and miR-484, were reported to target Tcl-1 in B-cell lymphoma (116). Finally, deregulated lncRNAs may also impact PI3K/Akt signaling. A study reported that the silencing of lncRNA HOX transcript antisense RNA (HOTAIR) in DLBCL cell lines led to increased cell apoptosis through reducing PI3K/Akt activation (56). lncRNA TUG1, which is overexpressed in DLBCL, affects PI3K/Akt signaling through interacting with MET and reducing its ubiquitination (48).

Other deregulated signaling pathways related to cell survival, such as mitogen-activated protein kinase (MAPK), NF-κB, and Wnt/β-catenin signaling pathways, have also been reported modulated by ncRNAs in B-cell lymphomas. For example, miR-101 was reported to target mitogen-activated protein kinase kinase 1 (MEK1), an upstream protein kinase of Erk, in DLBCL. The decreased level of miR-101 predicts poorer prognosis of DLBCL patients (43). lncRNA PANDA was found to negatively regulate MAPK/Erk signaling pathway in DLBCL cells (46). Downregulated PANDA resulted in decreased tumor cell apoptosis and increased cell proliferation. HOTAIR was also reported to activate MAPK/Erk signaling in B-cell lymphoma, which was mediated by its target miR-148b (176). A recent study showed miR-124, a tumor suppressor that is downregulated in DLBCL, was suppressed by NF-κB signaling. Previous studies have reported miR-124 suppressed cell survival and proliferation through targeting NF-κB in B-cell lymphomas, and thus miR-124 together with NF-κB formed an autoregulatory feedback loop (143, 177). Furthermore, several deregulated lncRNAs in B-cell lymphoma, including OR3A4, SMAD5-AS1, and FIRRE, were reported to modulate cell apoptosis and proliferation *via* Wnt/β-catenin signaling pathway (52, 54). Finally, circRNA-APC, which is downregulated in DLBCL, inhibits Wnt/β-catenin signaling through upregulating APC, a suppressor of β-catenin accumulation in the nucleus (58).

### ncRNAs Regulate the Cell Cycle in B-Cell Lymphoma

#### ncRNA Regulate Cell Cycle Regulatory Proteins

Cell-cycle progression is strictly controlled by numerous cell cycle regulatory proteins, including cyclin-dependent kinases (CDKs), CDK inhibitors (CKIs), cyclins, and members of the RB and E2F families. Dysregulation of these proteins may result in uncontrolled cell proliferation in B-cell lymphoma. miR-15a/16-1 modulates the expression of a series of proteins involved in G0/G1-S phase transition, including cyclin D1/2/3, cyclin E, and CDK 4/6 (110, 178). Decreased miR-15a/16-1 expression enhances activation of these proteins and consequently increases lymphoma cell proliferation *in vitro* and *in vivo*. In addition, miR-26a and miR-29 were reported to target CDK6 in MCL (80). Downregulation of miR-29 is identified as a prognostic marker of MCL patients. In addition, miR-26a was shown to directly target and inhibit EZH2 in BL, and thus modulates the expression of EZH2 targeted genes, including cyclin E2 and cyclin B1. Restored expression of miR-26a in BL cell lines leads to the arrested cell cycle and reduced cell proliferation (137). Overexpression of miR-221/222 promotes the G0/G1-S transition through modulating the CKI p27 in CLL (115). Additionally, the miR-17-92 cluster acts as a key player in lymphoma cell proliferation through regulating p21, a CKI family member that negatively regulates G1-S transition (82). Besides, miR-17, miR-20a, and miR-34a target and downregulate E2F transcription factor 1 (E2F1), a crucial determinant of the transition from G1 to S phase (113, 179).

Several lncRNAs were reported involved in the modulation of cell-cycle checkpoint proteins. The knockdown of MALAT1 in DLBCL and MCL cell lines leads to the arrested cell cycle (11, 180). Mechanistically, MALAT1 interacts with EZH2 and promotes methylation at the promoter of *p21* and *p27*, thus repressing expression of these two genes (11). Inhibition of lncRNA LUNAR1, which is highly expressed in DLBCL samples and patients, significantly arrests cell cycle, accompanied by downregulated E2F1 and cyclin D1, and upregulated p21 expression (57). The expression of lincRNA-p21 is significantly downregulated in DLBCL and is associated with the prognosis of DLBCL patients (50). Ectopic expression of lincRNA-p21 in DLBCL cells inhibits cell proliferation through modulating the expression of p21, cyclin D1, and CDK4 (50). In addition, lncRNA ROR1 antisense RNA 1 (ROR1-AS1), whose expression is significantly upregulated in MCL, downregulates p16 through interacting with EZH2 (87). Moreover, lncRNA MINCR is suggested to act as a modulator of c-Myc-mediated transcription of multiple genes involved in cell-cycle progression. The knockdown of MINCR in BL cell lines inhibits expression of these genes and consequently decreased cell proliferation (72).

#### ncRNAs Fine-Tune c-Myc Expression in B-Cell Lymphoma

Hyperactivity of c-Myc, which is associated with uncontrolled cell proliferation, is a hallmark of the highly aggressive B-cell lymphoma. ncRNAs may impact c-Myc expression in direct or indirect ways. For example, studies showed that the level of lncRNA growth arrest-specific 5 (GAS5) was negatively associated with c-Myc expression in DLBCL (181). Further analysis revealed that GAS5 modulated c-Myc expression at a level of translation, probably through its direct interaction with eukaryotic initiation factor 4F (eIF4F), a key factor of translation initiation complex (182). Similarly, lncRNA SNHG12 was reported to inhibit c-Myc translation in MCL, *via* interacting with eIF4F (84).

**Figure 3:**
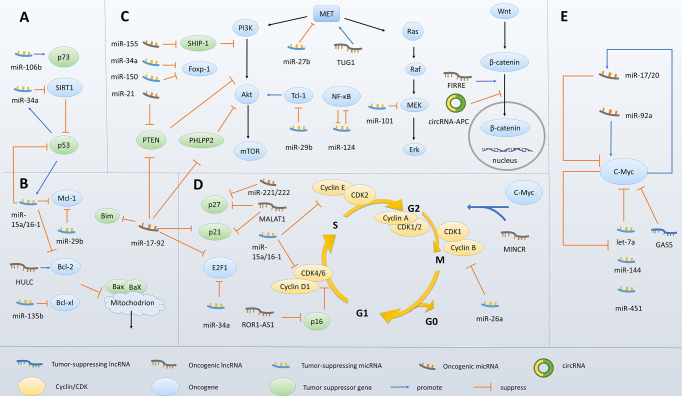
Molecular mechanisms underlying ncRNA-mediated survival and proliferation of malignant B cells. **(A)** ncRNAs modulate p53 expression. **(B)** ncRNAs modulate the Bcl-2 family members. **(C)** ncRNAs regulate key signaling pathways associated with cell survival. **(D)** ncRNAs modulate cell-cycle regulatory proteins. **(E)** ncRNAs fine-tune c-Myc expression.

Also, c-Myc induces dysregulation of numerous miRNAs, which in turn regulate its expression (39). let-7a is repressed by c-Myc, and let-7a targets c-Myc and inhibits its expression, suggesting that overexpressed c-Myc and let-7a form a feedback loop that stimulates c-Myc expression (105). As another example, miR-144/451 directly targets c-Myc and is inversely regulated by c-Myc at a level of transcription. Loss of miR-144/451 expression is able to initiate lymphoma-genesis in aged mice *via* c-Myc activation (183). In addition, miR-17-92 is upregulated by c-Myc in B-cell lymphomas (184). miR-92a upregulates c-Myc through suppressing FBW7 (185), a negative regulator of c-Myc, and thus forms a positive feedback loop. Interestingly, some c-Myc-induced ncRNAs, in turn, downregulate c-Myc expression. It was reported that miR-17/20 targeted Check2, whose downregulation induces the binding of HuR to the transcript of c-Myc, thus leading to inhibited c-Myc translation (136). These findings showed the role of ncRNAs in “fine-tuning” expression of c-Myc in B-cell lymphomas. On the one hand, the positive feedback loops of ncRNAs and c-Myc promote lymphomagenesis and progression. On the other hand, ncRNAs protect tumor cells from excessive c-Myc expression and consequent side effects, thus ensuring homeostasis of lymphoma cells (**Figure 3**).

#### ncRNAs Regulate Cell Differentiation in B-Cell Lymphoma

Given the role of ncRNAs in normal B-cell differentiation, it’s not surprising that dysregulated ncRNAs may lead to lymphomagenesis through altering B-cell differentiation processes. A study using Tet-off and Cre technology to generate mice with conditionally overexpressed miR-21 showed that the high level of miR-21 resulted in a pre-B malignant lymphoid-like phenotype (186). Further analysis showed that single cells from the lymph nodes of the miR-21-overexpressing mouse model represented undifferentiated immunotype. In contrast, cells from the wild-type control had the naïve and differentiated immunotypes (186). The finding suggested that miR-21 overexpression promotes lymphomagenesis, at least partly through impairing B-cell differentiation. Also, miR-127 is suggested to block B-cell differentiation through downregulating B lymphocyte-induced maturation protein-1 BLIMP-1, a master regulator of terminal B-cell differentiation. High expression of miR-127 in EBV-positive BL cells may explain the different morphology, immunophenotype, and gene expression between EBV-positive and -negative BL cells. Additionally, downregulated miR-150 by c-Myc is associated with the high grade transformation of FL, which is led by increased expression of miR-150 target FOXP1 (75). Ectopic expression of miR-150 in EBV-positive BL cell lines induced differentiation of the malignant GC B cells to the terminal stage through targeting c-Myb. Regarding that c-Myc-induced circRNA ZDHHC11 and ZDHHC11B downregulate miR-150 in BL (73), it can be hypothesized that dysregulation of these two circRNAs also impacts B-cell differentiation *via* sponging miR-150.

### ncRNA Mediate the Interaction Between Lymphoma Cells and the Microenvironment

Recent studies have described microenvironment as a critical player in tumor genesis and development (39). In B-cell lymphomas, the dynamic interactions between malignant cells and the lymphatic niches, including the release of chemokines, cytokines, and adhesion of B cells to the extracellular matrix, stromal cells, and other lymphocytes, are translated into intrinsic signaling pathways associated with cell survival, migration, and drug sensitivity. Of critical importance, some functional ncRNAs may be deregulated during the interaction, and some may influence the tumor microenvironment, such as angiogenesis and immune efficacy. Thus ncRNAs act as critical mediators in the interaction between B-cells and lymphoid microenvironment.

#### Cell-Cell Contact

Studies reported deregulated ncRNAs *via* cell-cell contact. The adhesion of malignant B cells to stromal follicular dendritic cells (FDCs) induces overexpression of miR-181a and subsequently decreased expression of its target Bim, which is associated with resistance of tumor cells to chemotherapies (76). Also, the adhesion of B cells to FDCs induces the downregulation of let-7 families and miR-9, and the upregulation of miR-30 family. Dysregulations of these miRNAs lead to increased expression of the transcription regulator PRDM1 and decreased expression of Bcl-6 (187). Moreover, the contact between lymphoma cells and stromal cells downregulates miR-548m, thus releasing its suppressive effect on HDAC6 (188). Decreased expression of miR-548m and increased expression of HDAC6 work synergistically to induce lymphoma formation and drug resistance. Finally, c-Myc was reported to be upregulated *via* the adhesion between malignant B cells and stromal cells. Considering that c-Myc regulates a series of ncRNAs in B cells, it can be hypothesized that increased expression of c-Myc *via* cell-cell contact contributes to ncRNA dysregulations in B-cell lymphomas. These findings together suggested that tumor microenvironment may induce lymphoma initiation, progression, and drug resistance through modulating ncRNA expression.

#### BCR Signaling

BCR acts as a key modulator of the interplay between extrinsic stimuli and intrinsic survival signaling cascades. Abnormal BCR signaling could trigger persistent activation of crucial signaling pathways associated with B-cell activation, proliferation, and migration, thus involved in the lymphoma onset and progression. Recent-year studies have revealed a correlation between ncRNA dysregulation and abnormal BCR signaling in B-cell lymphomas (172). miR-150 has been confirmed to target Grb2-associated binder 1 (GAB1) and Foxp1 in normal B cells and several B-cell lymphomas (23, 75, 75). Both GAB1 and Foxp1 positively regulate BCR signaling, as GAB1 promotes the recruitment of PI3K to the membrane, and Foxp1 transcriptionally activates genes involved in BCR signaling pathways (23). In addition to miR-150, miR-34a, and miR-181a were also found to target and regulate Foxp1 (141, 189). A recent study reported that miR-34a, which was upregulated by p53 during the activation of DNA repairing response, repressed Foxp1 and subsequently reduced the propensity of BCR signaling in CLL cells (141). This finding provides a new mechanism by which B cells stop the pro-proliferative signal when cell cycle arrest is required. Also, miR-155 is identified as a key player in BCR activation. Treating CLL cells with B-cell activators increases miR-155 expression and simultaneously enhances the sensitivity of BCR to its ligand (171). Further analysis suggested that the effect of miR-155 on BCR signaling was at least partly mediated by its target SHIP1, a negative regulator of PI3K/Akt and BCR activation (171). High expression of miR-17-92 cluster was also demonstrated to amplify BCR signaling through inhibiting CD22 and FCGR2B, which limit BCR activation through the immunoreceptor tyrosine inhibitory motifs (ITIM) (190). Additionally, a more recent study reported that miR-17-92 targeted PTPROT and PP2A, both of which are negative regulators of BCR signaling, suggesting another mechanism underlying miR-17-92-mediated BCR signaling deregulation (191). Moreover, miR-34b/c was confirmed to target zeta-associated protein 70 (ZAP-70), a protein that is supposed to lower the threshold of BCR signaling through enhancing phosphorylation of BCR signaling motifs (192). Finally, studies reported that miR-650 was encoded by the variable subgenes for IgL and upregulated when its host gene for IgL expressed. Given that miR-650 target genes are associated with cell proliferation such as CDK1, the regulation of miR-650 level is likely to help coordinate gene expression and B-cell biology with the expression of mature BCRs (193, 194). These findings showed that ncRNAs might mediate microenvironment-induced lymphomagenesis and development through modulating BCR, the central hub at the crossroad between extrinsic events and intrinsic signaling. Additionally, certain ncRNA expression can be triggered by BCR signaling. For example, the expression of miR-155 and its primary transcript is induced by the recruitment of JunB and FosB to miR-155 promotor after BCR activation, which is controlled by Erk and JNK signaling pathways (195).

#### Immune Modulation

Recent studies have shed light on the contribution of ncRNAs to tumor immune evasion. Overexpressed miR-155 in DLBCL cells leads to increased expression of programmed death-ligand 1 (PD-L1). Since PD-L1 induces Fas-mediated apoptosis and impairs the function of cytotoxic CD8+ T cells by interacting with PD-1, miR-155 is suggested to promote the immune evasion of lymphoma cells (196). A recent study reported EBV-encoded EBNA2 repressed miR-34a transcription, which in turn increased PD-L1 expression in lymphoma cells (151). As another example, lncRNA SNHG14 upregulates Zinc finger E-box binding homeobox 1 (ZEB1) through sponging miR-5590-3p. ZEB1 transcriptionally induces SNHG14 and PD-L1 expression. Thus SNHG14, ZEB1, and miR-5590-3p form a positive feedback loop that promotes PD-L1 expression (51).

**Figure 4:**
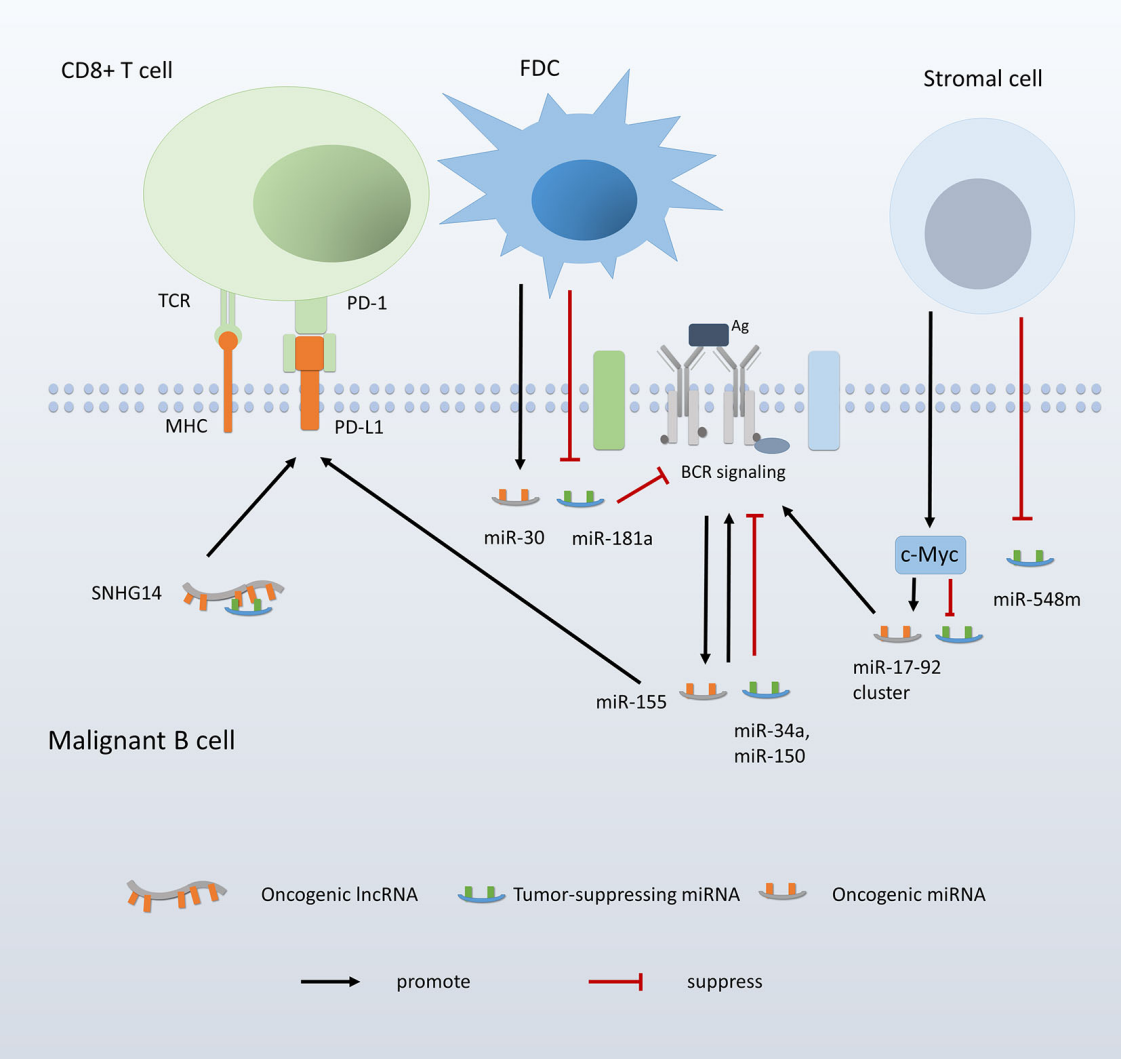
ncRNAs mediate the interaction between malignant B cells and the tumor environment.

Furthermore, the status of innate immune cells is also impacted by ncRNA expression. It was reported that miR-155 level was associated with macrophage polarization in the lymphoma microenvironment (197). Higher expression of miR-155 in EBV-negative DLBCL samples was suggested to related to a lower ratio of M2 macrophages, which plays an immunosuppressive role in the tumor environment. This finding is consistent with a previous study in which miR-155-knockdown accelerated tumor growth through impairing classic activation of M1 and skewing the macrophages toward an M2 phenotype (198) (**Figure 4**).

### Therapeutic Potential of ncRNA in B-Cell Lymphoma

Strategies for targeting ncRNAs vary depending on ncRNA subtype, subcellular location, and whether they act as suppressor or promotor during malignant transformation.

#### miRNA-Targeted Therapeutics

miRNA-targeted therapies include two major therapeutic approaches depending on their function and aberrant status in malignancies. The first one is miRNA replacement, which aims to enforce the expression of tumor suppressor miRNAs. It could be achieved by delivering small synthetic RNA duplex, which mimics endogenous miRNAs. The therapeutic effects of certain tumor-suppressor miRNAs have been tested in B-lymphoma preclinical models. For example, prophylactic and therapeutic delivery of miR-28 through viral vectors showed significant antitumor effects in DLBCL and BL xenografts, and in murine primary lymphoma models (33). The other approach of miRNA-targeted therapy is inhibition of tumor-inducing miRNAs, which is mainly based on the application of antisense single-stranded oligonucleotides (ASOs). ASOs function through binding to the complementary RNA and silencing the target RNA through endonucleolytic cleavage. Various chemical modifications have been developed to increase their stability for *in vivo* delivery, such as locked nucleic acid (LNA) oligonucleotides, cholesterol-conjugated antagomirs, and polylysine-conjugated peptide nucleic acids (199). Some anti-miRNA agents have been tested in B-cell lymphomas, such as LNA-anti-miR-155, which showed a significant effect in the murine model (200). Besides, an oligonucleotide named Cobomarsen, which targets miR-155, is being tested in phase 1 clinical trial for patients with cutaneous T-cell lymphoma (201). Compared with miRNA mimics, anti-miRNA therapy has shown less off-target effects in preclinical trials, and more clinical testing is needed to evaluate the dosing schedule, efficacy, side effects, and toxicity of these therapeutics.

#### lncRNA-Targeted Therapeutics

Double-stranded RNA-mediated interference (RNAi) and ASOs are commonly used in lncRNA-targeted therapies. RNAi is mediated by short antisense RNA through binding to its target lncRNA and inducing RISC-mediated lncRNA silencing and degradation. However, the effect is limited in cases that the target lncRNA has extensive secondary structure or localizes in the nucleus, and it’s mandatory to use ASOs in such conditions. The above-described chemical modifications are also applied to increase the stability and specificity of lncRNA-targeting ASOs. LNA enhanced antisense oligonucleotides (LNA-ASO), for example, have shown optimal effects, particularly for lncRNAs located in the nucleus. LNA-ASO could be directly used for *in vivo* delivery. The remarkable effect and low toxicity of LNA-ASO targeting lncRNAs have been reported in hematological malignancies. For example, Amodio et al. showed that naked delivery of MALAT1-targeted LNA‐gapmeR (a modified ASO) significantly knocked down MALAT1 in MM cells, with a consequently reduced cell proliferation and bortezomib resistance both *in vitro* and *in vivo* (202). Regarding the roles of oncogenic lncRNAs in lymphomagenesis and progression, relative researches may be promising in B-cell lymphomas.

## Conclusions and future perspectives

Current evidence reveals that numerous ncRNAs are deregulated in B-cell lymphoma, and it’s interesting to observe that a number of dysregulated ncRNAs act as key regulators during normal B-cell development, which suggests the link between the physiological and pathological role of ncRNAs in B cells. Despite the heterogeneous biologic nature of B-cell lymphomas, many ncRNAs are frequently dysregulated in different lymphoma subtypes, as a result of genetic and epigenetic, transcriptional, and posttranscriptional abnormalities. Deregulated ncRNAs play an essential role in regulating cell survival, proliferation, and differentiation, as well as mediating the interaction between malignant B cells and the tumor environment, and thus promote lymphoma onset and progression. Given that ncRNAs are involved in various oncogenetic processes, studies about ncRNA expression and function are important for the understanding of tumor biology and the development of ncRNA-targeted therapy. However, despite the significant progress achieved so far, the functions and regulatory mechanisms of most lncRNAs and circRNAs remain elusive. There are few studies about the role of the secondary structure of lncRNAs in tumors, which are crucial for the biological function of lncRNAs (203). Recent studies also highlighted the role of RNA modifications, including m6A modification in ncRNAs. m6A modification is associated with structural changes on lncRNAs (204), and these changes may impact the interaction between lncRNAs and their binding partners (205), thus regulating the function and stability of lncRNAs. Another concern is that current knowledge of the function and regulatory mechanism of ncRNAs is fragmented across cancer types. Some ncRNAs have been reported to function as a tumor suppressor or oncogene in different tumors, and the regulatory mechanisms may be influenced by the cellular context. The fragmented information about ncRNA functions may be an issue in such conditions. In addition, compared with miRNAs and lncRNAs, the study of circRNA expression can be more challenging. An important issue is that most public RNA-seq datasets do not contain the information of circRNA, as a result of the traditional RNA library preparation, during which RNAs without poly-A tails are removed.

ncRNA expression profiling has the potential for lymphoma diagnosis. As we have mentioned, the ncRNA expression signature may help distinguish different lymphoma entities and optimize the standard of lymphoma classification, which keeps evolving with the improving understanding of the molecular nature of lymphoma. Besides, certain ncRNAs are correlated with lymphoma progression or drug resistance, suggesting that stratification of patients based on ncRNA expression may be a promising tool to predict prognosis and improve future management of B-cell lymphoma patients. To note that, increasingly research has focused on the expression pattern of ncRNAs in biological fluids, which are called circulating ncRNAs. It’s shown that differential expression of circulating ncRNAs could help distinguish B-cell lymphoma patients, suggesting that the method of ncRNA-based diagnosis or stratification may be achieved in a non-invasive way. For example, a recent study showed a serum-miRNA signature, including miR-18a, miR-24, miR-15a, and let7b/c, to distinguish DLBCL patients from normal controls (206). As another example, serum lncRNA RP11-513G11.1 was reported to be highly expressed in DLBCL, and a higher level of serum RP11-513G11.1 was associated with shorter PFS and OS of DLBCL patients (207). Though detecting the expression of circulating ncRNAs has been considered as a promising method for lymphoma diagnosis and stratification, there are still some challenges, including the high variability and low yield of RNA extracted from the serum or plasma. The optimization of serum/plasma RNA purification and the normalization of data processing are needed in the future.

Considering that ncRNAs participate in nearly every aspect of lymphoma pathogenesis, it’s promising to treat ncRNA as therapeutic targets in B-cell lymphoma. However, the field is still in its infancy stage, with relatively insufficient data and some unsolved issues. One major concern is how to improve the specificity and efficiency of targeting specific ncRNAs in the malignant cells. Another problem is that the ncRNA regulatory network is complex and hasn’t been fully understood, and thus may cause unexpected results during preclinical application. For example, a study reported that enforced expression of tumor suppressor miR-34a in B-cell lymphoma cells confers bortezomib resistance through inhibiting bortezomib-induced cell apoptosis (138). So far, the majority of lncRNAs and circRNAs haven’t been studied yet, which limits their therapeutic potential, and more basic research is needed to clarify their functions as well as regulatory mechanisms.

Despite the above-described challenges, we can expect the combination of ncRNA-based therapeutics and other approaches in the future. ncRNA dysregulations are tightly correlated with chemoresistance and radioresistance across human cancers (208). The application of ncRNA-targeted therapy has the potential to reverse such resistance of tumor cells. Additionally, recent studies have shed light on chimeric antigen receptor (CAR) T cell immunotherapy, the novel type of adoptive cell transfer therapeutics, which has been approved by the Food and Drug Administration for treatment in advanced B-cell lymphoma. Considering that ncRNAs contribute to the regulation of tumor immunity, including T-cell responses, it’s promising to combine CAR T therapies with ncRNA-based therapeutics to improve treatment efficacy, which has been validated by several preclinical studies (209, 210).

## Author Contributions

JL and JZ wrote the manuscript and created the figures. XW, CS, and FP made revisions to the manuscript. ZC and YH provided direction and guidance throughout the preparation of this manuscript. All authors contributed to the article and approved the submitted version.

## Funding

This study was supported by The National Natural Science Foundation of China (81302042, 81400148).

## Conflict of Interest

The authors declare that the research was conducted in the absence of any commercial or financial relationships that could be construed as a potential conflict of interest.

## Abbreviations

AID, Activation-induced cytidine deaminase; Akt, Protein kinase B; ASO, Antisense single-stranded oligonucleotide; Bcl-2, B-cell lymphoma-2; BCR, B-cell receptor; BL, Burkitt lymphoma; BM, Bone marrow; CAR, Chimeric antigen receptor; CDK, Cyclin-dependent kinase; CKI, CDK inhibitor; CLL, chronic lymphocytic leukemia; CRNDE, Colorectal neoplasia differentially expressed; circRNA, circular RNA; DLBCL, Diffuse large B-cell lymphoma; E2F1, E2F transcription factor 1; EBNA2, Epstein-Barr nuclear antigen 2; eIF4F, Eukaryotic initiation factor 4F; EZH2, Enhancer of zeste homolog 2; FAS-AS1, FAS antisense RNA 1; FDC, Follicular dendritic cell; FIRRE, Functional intergenic repeating RNA element; FL, Follicular lymphoma; Foxp1, Forkhead box P1; GAB1, Grb2-associated binder 1; GAS5, Growth arrest-specific 5; H3K27me3, Trimethylation of lysine 27 on histone 3; HOTAIR, HOX transcript antisense RNA; HULC, Highly upregulated in liver cancer; Ig, Immunoglobulin; LMP1, Latent membrane protein-1; lncRNA, long noncoding RNA; lincRNA-p21, long intergenic noncoding RNA p21; LUNAR1: leukemia-associated noncoding IGF1R activator RNA 1; MALAT1, Metastasis-associated lung adenocarcinoma transcript 1; MAPK, Mitogen-activated protein kinase; MCL, Mantle cell lymphoma; Mcl-1, Myeloid cell leukemia-1; MINCR, Myc-induced long noncoding RNA; miRNA, microRNA; ncRNA, Non-coding RNA; NEAT1, Nuclear enriched abundant transcript 1; PANDA, p21-associated ncRNA DNA damage activated; PD-L1, Programmed death ligand-1; PI3K, Phosphoinositide 3-kinase; PRDM1, Transcription regulator PR domain containing 1; PTEN, Protein tyrosine phosphatase; RISC, RNA-induced silencing complex; ROR1-AS1, ROR1 antisense RNA 1; S1PR1, Sphingosine-1-phosphate receptor 1; SHIP-1, SH2-containing inositol phosphatase-1; SNHG12, Small nucleolar RNA host gene 12; Tcl1, T-cell leukemia/lymphoma 1; TUG1, Taurine upregulated 1.

## References

[1] Dunham I Kundaje A Aldred SF Collins PJ Davis CA Doyle F An integrated encyclopedia of DNA elements in the human genome. Nature (2012) 489(7414):57–74. 10.1038/nature11247 22955616 PMC3439153

[2] Calin GA Dumitru CD Shimizu M Bichi R Zupo S Noch E Frequent deletions and down-regulation of micro- RNA genes miR15 and miR16 at 13q14 in chronic lymphocytic leukemia. Proc Natl Acad Sci USA (2002) 99(24):15524–9. 10.1073/pnas.242606799 PMC137750 12434020

[3] Drusco A Croce CM MicroRNAs and Cancer: A Long Story for Short RNAs. Adv Cancer Res (2017) 135:1–24. 10.1016/bs.acr.2017.06.005 28882219

[4] Eulalio A Huntzinger E Izaurralde E Getting to the root of miRNA-mediated gene silencing. Cell (2008) 132(1):9–14. 10.1016/j.cell.2007.12.024 18191211

[5] Kopp F Mendell JT Functional Classification and Experimental Dissection of Long Noncoding RNAs. Cell (2018) 172(3):393–407. 10.1016/j.cell.2018.01.011 29373828 PMC5978744

[6] Long J Badal SS Ye Z Wang Y Ayanga BA Galvan DL Long noncoding RNA Tug1 regulates mitochondrial bioenergetics in diabetic nephropathy. J Clin Invest (2016) 126(11):4205–18. 10.1172/JCI87927 PMC5096930 27760051

[7] Chédin F Nascent Connections: R-Loops and Chromatin Patterning. Trends Genet (2016) 32(12):828–38. 10.1016/j.tig.2016.10.002 PMC5123964 27793359

[8] Latos PA Pauler FM Koerner MV Şenergin HB Hudson QJ Stocsits RR Airn transcriptional overlap, but not its lncRNA products, induces imprinted Igf2r silencing. Science (2012) 338(6113):1469–72. 10.1126/science.1228110 23239737

[9] Romero-Barrios N Legascue MF Benhamed M Ariel F Crespi M Splicing regulation by long noncoding RNAs. Nucleic Acids Res (2018) 46(5):2169–84. 10.1093/nar/gky095 PMC5861421 29425321

[10] Lu Y Liu X Xie M Liu M Ye M Li M The NF-κB-Responsive Long Noncoding RNA FIRRE Regulates Posttranscriptional Regulation of Inflammatory Gene Expression through Interacting with hnRNPU. J Immunol (2017) 199(10):3571–82. 10.4049/jimmunol.1700091 PMC5672816 28993514

[11] Wang X Sehgal L Jain N Khashab T Mathur R Samaniego F LncRNA MALAT1 promotes development of mantle cell lymphoma by associating with EZH2. J Transl Med (2016) 14(1):346. 10.1186/s12967-016-1100-9 27998273 PMC5175387

[12] Gutschner T Diederichs S The hallmarks of cancer: a long non-coding RNA point of view. RNA Biol (2012) 9(6):703–19. 10.4161/rna.20481 PMC3495743 22664915

[13] Conn SJ Pillman KA Toubia J Conn VM Salmanidis M Phillips CA The RNA binding protein quaking regulates formation of circRNAs. Cell (2015) 160(6):1125–34. 10.1016/j.cell.2015.02.014 25768908

[14] Memczak S Jens M Elefsinioti A Torti F Krueger J Rybak A Circular RNAs are a large class of animal RNAs with regulatory potency. Nature (2013) 495(7441):333–8. 10.1038/nature11928 23446348

[15] Hentze MW Preiss T Circular RNAs: splicing’s enigma variations. EMBO J (2013) 32(7):923–5. 10.1038/emboj.2013.53 PMC3616293 23463100

[16] Pamudurti NR Bartok O Jens M Ashwal-Fluss R Stottmeister C Ruhe L Translation of CircRNAs. Mol Cell (2017) 66(1):9–21.e7. 10.1016/j.molcel.2017.02.021 28344080 PMC5387669

[17] Liu P Li X Cui Y Chen J Li C Li Q LncRNA-MALAT1 mediates cisplatin resistance via miR-101-3p/VEGF-C pathway in bladder cancer. Acta Biochim Biophys Sin (Shanghai) (2019) 51(11):1148–57. 10.1093/abbs/gmz112 31650173

[18] Zhang Y Wang F Chen G He R Yang L LncRNA MALAT1 promotes osteoarthritis by modulating miR-150-5p/AKT3 axis. Cell Biosci (2019) 9:54. 10.1186/s13578-019-0302-2 31304004 PMC6600894

[19] Zheng J Huang X Tan W Yu D Du Z Chang J Pancreatic cancer risk variant in LINC00673 creates a miR-1231 binding site and interferes with PTPN11 degradation. Nat Genet (2016) 48(7):747–57. 10.1038/ng.3568 27213290

[20] Chen CZ Li L Lodish HF Bartel DP MicroRNAs modulate hematopoietic lineage differentiation. Science (2004) 303(5654):83–6. 10.1126/science.1091903 14657504

[21] Ventura A Young AG Winslow MM Lintault L Meissner A Erkeland SJ Targeted deletion reveals essential and overlapping functions of the miR-17 through 92 family of miRNA clusters. Cell (2008) 132(5):875–86. 10.1016/j.cell.2008.02.019 PMC2323338 18329372

[22] Rao DS O'Connell RM Chaudhuri AA Garcia-Flores Y Geiger TL Baltimore D MicroRNA-34a perturbs B lymphocyte development by repressing the forkhead box transcription factor Foxp1. Immunity (2010) 33(1):48–59. 10.1016/j.immuni.2010.06.013 20598588 PMC2911227

[23] Mraz M Chen L Rassenti LZ Ghia EM Li H Jepsen K miR-150 influences B-cell receptor signaling in chronic lymphocytic leukemia by regulating expression of GAB1 and FOXP1. Blood (2014) 124(1):84–95. 10.1182/blood-2013-09-527234 24787006 PMC4125356

[24] Xiao C Calado DP Galler G Thai TH Patterson HC Wang J MiR-150 controls B cell differentiation by targeting the transcription factor c-Myb. Cell (2007) 131(1):146–59. 10.1016/j.cell.2007.07.021 17923094

[25] Tan LP Wang M Robertus JL Schakel RN Gibcus JH Diepstra A miRNA profiling of B-cell subsets: specific miRNA profile for germinal center B cells with variation between centroblasts and centrocytes. Lab Invest (2009) 89(6):708–16. 10.1038/labinvest.2009.26 19349957

[26] Zhou B Wang S Mayr C Bartel DP Lodish HF miR-150, a microRNA expressed in mature B and T cells, blocks early B cell development when expressed prematurely. Proc Natl Acad Sci USA (2007) 104(17):7080–5. 10.1073/pnas.0702409104 PMC1855395 17438277

[27] Mehta A Mann M Zhao JL Marinov GK Majumdar D Garcia-Flores Y The microRNA-212/132 cluster regulates B cell development by targeting Sox4. J Exp Med (2015) 212(10):1679–92. 10.1084/jem.20150489 PMC4577845 26371188

[28] Li G So AY Sookram R Wong S Wang JK Ouyang Y Epigenetic silencing of miR-125b is required for normal B-cell development. Blood (2018) 131(17):1920–30. 10.1182/blood-2018-01-824540 PMC5921965 29555645

[29] Vigorito E Perks KL Abreu-Goodger C Bunting S Xiang Z Kohlhaas S microRNA-155 regulates the generation of immunoglobulin class-switched plasma cells. Immunity (2007) 27(6):847–59. 10.1016/j.immuni.2007.10.009 PMC4135426 18055230

[30] Thai TH Calado DP Casola S Ansel KM Xiao C Xue Y Regulation of the germinal center response by microRNA-155. Science (2007) 316(5824):604–8. 10.1126/science.1141229 17463289

[31] Teng G Hakimpour P Landgraf P Rice A Tuschl T Casellas R MicroRNA-155 is a negative regulator of activation-induced cytidine deaminase. Immunity (2008) 28(5):621–9. 10.1016/j.immuni.2008.03.015 PMC2430982 18450484

[32] de Yébenes VG Belver L Pisano DG González S Villasante A Croce C miR-181b negatively regulates activation-induced cytidine deaminase in B cells. J Exp Med (2008) 205(10):2199–206. 10.1084/jem.20080579 PMC2556787 18762567

[33] Bartolomé-Izquierdo N de Yébenes VG Álvarez-Prado AF Mur SM Lopez Del Olmo JA Roa S miR-28 regulates the germinal center reaction and blocks tumor growth in preclinical models of non-Hodgkin lymphoma. Blood (2017) 129(17):2408–19. 10.1182/blood-2016-08-731166 PMC5437734 28188132

[34] de Yébenes VG Bartolomé-Izquierdo N Nogales-Cadenas R Pérez-Durán P Mur SM Martínez N miR-217 is an oncogene that enhances the germinal center reaction. Blood (2014) 124(2):229–39. 10.1182/blood-2013-12-543611 24850757

[35] Petri A Dybkær K Bøgsted M Thrue CA Hagedorn PH Schmitz A Long Noncoding RNA Expression during Human B-Cell Development. PloS One (2015) 10(9):e0138236. 10.1371/journal.pone.0138236 26394393 PMC4578992

[36] Ellis BC Graham LD Molloy PL CRNDE, a long non-coding RNA responsive to insulin/IGF signaling, regulates genes involved in central metabolism. Biochim Biophys Acta (2014) 1843(2):372–86. 10.1016/j.bbamcr.2013.10.016 24184209

[37] Ebralidze AK Guibal FC Steidl U Zhang P Lee S Bartholdy B PU.1 expression is modulated by the balance of functional sense and antisense RNAs regulated by a shared cis-regulatory element. Genes Dev (2008) 22(15):2085–92. 10.1101/gad.1654808 PMC2492744 18676813

[38] DeKoter RP Singh H Regulation of B lymphocyte and macrophage development by graded expression of PU.1. Science (2000) 288(5470):1439–41. 10.1126/science.288.5470.1439 10827957

[39] Musilova K Mraz M MicroRNAs in B-cell lymphomas: how a complex biology gets more complex. Leukemia (2015) 29(5):1004–17. 10.1038/leu.2014.351 25541152

[40] Caramuta S Lee L Ozata DM Akçakaya P Georgii-Hemming P Xie H Role of microRNAs and microRNA machinery in the pathogenesis of diffuse large B-cell lymphoma. Blood Cancer J (2013) 3(10):e152. 10.1038/bcj.2013.49 24121164 PMC3816210

[41] Roehle A Hoefig KP Repsilber D Thorns C Ziepert M Wesche KO MicroRNA signatures characterize diffuse large B-cell lymphomas and follicular lymphomas. Br J Haematol (2008) 142(5):732–44. 10.1111/j.1365-2141.2008.07237.x 18537969

[42] Iqbal J Shen Y Huang X Liu Y Wake L Liu C Global microRNA expression profiling uncovers molecular markers for classification and prognosis in aggressive B-cell lymphoma. Blood (2015) 125(7):1137–45. 10.1182/blood-2014-04-566778 PMC4326773 25498913

[43] Huang Y Zou Y Lin L Ma X Zheng R miR−101 regulates the cell proliferation and apoptosis in diffuse large B−cell lymphoma by targeting MEK1 via regulation of the ERK/MAPK signaling pathway. Oncol Rep (2019) 41(1):377–86. 10.3892/or.2018.6821 30365139

[44] Huang Y Zou Y Lin L Ma X Zheng R miR-101 regulates cell proliferation and apoptosis by targeting KDM1A in diffuse large B cell lymphoma. Cancer Manag Res (2019) 11:2739–46. 10.2147/CMAR.S197744 PMC6455001 31040714

[45] Kozloski GA Jiang X Bhatt S Ruiz J Vega F Shaknovich R miR-181a negatively regulates NF-κB signaling and affects activated B-cell-like diffuse large B-cell lymphoma pathogenesis. Blood (2016) 127(23):2856–66. 10.1182/blood-2015-11-680462 26941399

[46] Zhou M Zhao H Xu W Bao S Cheng L Sun J Discovery and validation of immune-associated long non-coding RNA biomarkers associated with clinically molecular subtype and prognosis in diffuse large B cell lymphoma. Mol Cancer (2017) 16(1):16. 10.1186/s12943-017-0580-4 28103885 PMC5248456

[47] Zhao C-C Jiao Y Zhang YY Ning J Zhang YR Xu J Lnc SMAD5-AS1 as ceRNA inhibit proliferation of diffuse large B cell lymphoma via Wnt/β-catenin pathway by sponging miR-135b-5p to elevate expression of APC. Cell Death Dis (2019) 10(4):252. 10.1038/s41419-019-1479-3 30874550 PMC6420660

[48] Cheng H Yan Z Wang X Cao J Chen W Qi K Downregulation of long non-coding RNA TUG1 suppresses tumor growth by promoting ubiquitination of MET in diffuse large B-cell lymphoma. Mol Cell Biochem (2019) 461(1):47–56. 10.1007/s11010-019-03588-7 31338678

[49] Sehgal L Mathur R Braun FK Wise JF Berkova Z Neelapu S FAS-antisense 1 lncRNA and production of soluble versus membrane Fas in B-cell lymphoma. Leukemia (2014) 28(12):2376–87. 10.1038/leu.2014.126 PMC5827933 24811343

[50] Peng W Wu J Feng J LincRNA-p21 predicts favorable clinical outcome and impairs tumorigenesis in diffuse large B cell lymphoma patients treated with R-CHOP chemotherapy. Clin Exp Med (2017) 17(1):1–8. 10.1007/s10238-015-0396-8 26475621

[51] Zhao L Liu Y Zhang J Liu Y Qi Q LncRNA SNHG14/miR-5590-3p/ZEB1 positive feedback loop promoted diffuse large B cell lymphoma progression and immune evasion through regulating PD-1/PD-L1 checkpoint. Cell Death Dis (2019) 10(10):731. 10.1038/s41419-019-1886-5 31570691 PMC6769008

[52] Meng H Zhao B Wang Y FOXM1-induced upregulation of lncRNA OR3A4 promotes the progression of diffuse large B-cell lymphoma via Wnt/β-catenin signaling pathway. Exp Mol Pathol (2020) 115:104451. 10.1016/j.yexmp.2020.104451 32417392

[53] Qian C-S Li LJ Huang HW Yang HF Wu DP MYC-regulated lncRNA NEAT1 promotes B cell proliferation and lymphomagenesis via the miR-34b-5p-GLI1 pathway in diffuse large B-cell lymphoma. Cancer Cell Int (2020) 20(1):87. 10.1186/s12935-020-1158-6 32206038 PMC7081629

[54] Shi X Cui Z Liu X Wu S Wu Y Fang F LncRNA FIRRE is activated by MYC and promotes the development of diffuse large B-cell lymphoma via Wnt/β-catenin signaling pathway. Biochem Biophys Res Commun (2019) 510(4):594–600. 10.1016/j.bbrc.2019.01.105 30739786

[55] Peng W Wu J Feng J Long noncoding RNA HULC predicts poor clinical outcome and represents pro-oncogenic activity in diffuse large B-cell lymphoma. BioMed Pharmacother (2016) 79:188–93. 10.1016/j.biopha.2016.02.032 27044827

[56] Yan Y Han J Li Z Yang H Sui Y Wang M Elevated RNA expression of long non−coding HOTAIR promotes cell proliferation and predicts a poor prognosis in patients with diffuse large B cell lymphoma. Mol Med Rep (2016) 13(6):5125–31. 10.3892/mmr.2016.5190 PMC4878541 27122348

[57] Peng W Feng J Long noncoding RNA LUNAR1 associates with cell proliferation and predicts a poor prognosis in diffuse large B-cell lymphoma. BioMed Pharmacother (2016) 77:65–71. 10.1016/j.biopha.2015.12.001 26796267

[58] Hu Y Zhao Y Shi C Ren P Wei B Guo Y A circular RNA from APC inhibits the proliferation of diffuse large B-cell lymphoma by inactivating Wnt/β-catenin signaling via interacting with TET1 and miR-888. Aging (Albany NY) (2019) 11(19):8068–84. 10.18632/aging.102122 PMC6814595 31631067

[59] Fulci V Chiaretti S Goldoni M Azzalin G Carucci N Tavolaro S Quantitative technologies establish a novel microRNA profile of chronic lymphocytic leukemia. Blood (2007) 109(11):4944–51. 10.1182/blood-2006-12-062398 17327404

[60] Zenz T Mohr J Eldering E Kater AP Bühler A Kienle D miR-34a as part of the resistance network in chronic lymphocytic leukemia. Blood (2009) 113(16):3801–8. 10.1182/blood-2008-08-172254 18941118

[61] Kaur G Ruhela V Rani L Gupta A Sriram K Gogia A RNA-Seq profiling of deregulated miRs in CLL and their impact on clinical outcome. Blood Cancer J (2020) 10(1):6. 10.1038/s41408-019-0272-y 31932582 PMC6957689

[62] Vasyutina E Boucas JM Bloehdorn J Aszyk C Crispatzu G Stiefelhagen M The regulatory interaction of EVI1 with the TCL1A oncogene impacts cell survival and clinical outcome in CLL. Leukemia (2015) 29(10):2003–14. 10.1038/leu.2015.114 25936528

[63] Kopparapu PK Bhoi S Mansouri L Arabanian LS Plevova K Pospisilova S Epigenetic silencing of miR-26A1 in chronic lymphocytic leukemia and mantle cell lymphoma: Impact on EZH2 expression. Epigenetics (2016) 11(5):335–43. 10.1080/15592294.2016.1164375 PMC4889270 27052808

[64] Due H Svendsen P Bødker JS Schmitz A Bøgsted M Johnsen HE miR-155 as a Biomarker in B-Cell Malignancies. BioMed Res Int (2016) 2016:9513037. 10.1155/2016/9513037 27294145 PMC4884835

[65] Subhash S Andersson PO Kosalai ST Kanduri C Kanduri M Global DNA methylation profiling reveals new insights into epigenetically deregulated protein coding and long noncoding RNAs in CLL. Clin Epigenet (2016) 8:106. 10.1186/s13148-016-0274-6 PMC5062931 27777635

[66] Xia L Wu L Bao J Li Q Chen X Xia H Circular RNA circ-CBFB promotes proliferation and inhibits apoptosis in chronic lymphocytic leukemia through regulating miR-607/FZD3/Wnt/β-catenin pathway. Biochem Biophys Res Commun (2018) 503(1):385–90. 10.1016/j.bbrc.2018.06.045 29902450

[67] Dzikiewicz-Krawczyk A Diepstra A Rutgers B Kortman G de Jong D Koerts J Argonaute 2 RNA Immunoprecipitation Reveals Distinct miRNA Targetomes of Primary Burkitt Lymphoma Tumors and Normal B Cells. Am J Pathol (2018) 188(5):1289–99. 10.1016/j.ajpath.2018.01.018 29458013

[68] Kluiver J Elton TS Selemon H Elton SM Parinandi NL Regulation of pri-microRNA BIC transcription and processing in Burkitt lymphoma. Oncogene (2007) 26(26):3769–76. 10.1038/sj.onc.1210147 17173072

[69] Mazzoccoli L Robaina MC Apa AG Bonamino M Pinto LW Queiroga E MiR-29 silencing modulates the expression of target genes related to proliferation, apoptosis and methylation in Burkitt lymphoma cells. J Cancer Res Clin Oncol (2018) 144(3):483–97. 10.1007/s00432-017-2575-3 PMC11813525 29318382

[70] Di Lisio L Sánchez-Beato M Gómez-López G Rodríguez ME Montes-Moreno S Mollejo M MicroRNA signatures in B-cell lymphomas. Blood Cancer J (2012) 2(2):e57–7. 10.1038/bcj.2012.1 PMC3288280 22829247

[71] Han B Wang S Zhao H MicroRNA-21 and microRNA-155 promote the progression of Burkitt’s lymphoma by the PI3K/AKT signaling pathway. Int J Clin Exp Pathol (2020) 13(1):89–98. 32055277 PMC7013371

[72] Doose G Haake A Bernhart SH López C Duggimpudi S Wojciech F MINCR is a MYC-induced lncRNA able to modulate MYC’s transcriptional network in Burkitt lymphoma cells. Proc Natl Acad Sci USA (2015) 112(38):E5261–70. 10.1073/pnas.1505753112 PMC4586867 26351698

[73] Dzikiewicz-Krawczyk A Kok K Slezak-Prochazka I Robertus JL Bruining J Tayari MM ZDHHC11 and ZDHHC11B are critical novel components of the oncogenic MYC-miR-150-MYB network in Burkitt lymphoma. Leukemia (2017) 31(6):1470–3. 10.1038/leu.2017.94 28331227

[74] Leich E Zamo A Horn H Haralambieva E Puppe B Gascoyne RD MicroRNA profiles of t(14;18)-negative follicular lymphoma support a late germinal center B-cell phenotype. Blood (2011) 118(20):5550–8. 10.1182/blood-2011-06-361972 PMC3217356 21960592

[75] Musilova K Devan J Cerna K Seda V Pavlasova G Sharma S miR-150 downregulation contributes to the high-grade transformation of follicular lymphoma by upregulating FOXP1 levels. Blood (2018) 132(22):2389–400. 10.1182/blood-2018-06-855502 30213873

[76] Lwin T Lin J Choi YS Zhang X Moscinski LC Wright KL Follicular dendritic cell-dependent drug resistance of non-Hodgkin lymphoma involves cell adhesion-mediated Bim down-regulation through induction of microRNA-181a. Blood (2010) 116(24):5228–36. 10.1182/blood-2010-03-275925 PMC3012540 20841506

[77] Wang W Corrigan-Cummins M Hudson J Maric I Simakova O Neelapu SS MicroRNA profiling of follicular lymphoma identifies microRNAs related to cell proliferation and tumor response. Haematologica (2012) 97(4):586–94. 10.3324/haematol.2011.048132 PMC3347661 22102710

[78] Pan Y Guo Y Luo Y Li H Xu Y MicroRNA expression profiling of Chinese follicular lymphoma by microarray: A preliminary study. Int Immunopharmacol (2016) 39:41–7. 10.1016/j.intimp.2016.07.006 27409728

[79] Arribas AJ Campos-Martín Y Gómez-Abad C Algara P Sánchez-Beato M Rodriguez-Pinilla MS Nodal marginal zone lymphoma: gene expression and miRNA profiling identify diagnostic markers and potential therapeutic targets. Blood (2012) 119(3):e9–e21. 10.1182/blood-2011-02-339556 22110251

[80] Zhao JJ Lin J Lwin T Yang H Guo J Kong W microRNA expression profile and identification of miR-29 as a prognostic marker and pathogenetic factor by targeting CDK6 in mantle cell lymphoma. Blood (2010) 115(13):2630–9. 10.1182/blood-2009-09-243147 PMC2852365 20086245

[81] Rao E Jiang C Ji M Huang X Iqbal J Lenz G The miRNA-17∼92 cluster mediates chemoresistance and enhances tumor growth in mantle cell lymphoma via PI3K/AKT pathway activation. Leukemia (2012) 26(5):1064–72. 10.1038/leu.2011.305 22116552

[82] Inomata M Tagawa H Guo YM Kameoka Y Takahashi N Sawada K MicroRNA-17-92 down-regulates expression of distinct targets in different B-cell lymphoma subtypes. Blood (2009) 113(2):396–402. 10.1182/blood-2008-07-163907 18941111

[83] Iqbal J Shen Y Liu Y Fu K Jaffe ES Liu C Genome-wide miRNA profiling of mantle cell lymphoma reveals a distinct subgroup with poor prognosis. Blood (2012) 119(21):4939–48. 10.1182/blood-2011-07-370122 PMC3367895 22490335

[84] Hu G Zhang Y Gupta M RIP sequencing in mantle cell lymphoma identifies functional long non-coding RNAs associated with translation machinery. Blood Cancer J (2019) 9(8):55. 10.1038/s41408-019-0216-6 31350385 PMC6659685

[85] Mu G Liu Q Wu S Xia Y Fang Q Long noncoding RNA HAGLROS promotes the process of mantle cell lymphoma by regulating miR-100/ATG5 axis and involving in PI3K/AKT/mTOR signal. Artif Cells Nanomedicine Biotechnol (2019) 47(1):3649–56. 10.1080/21691401.2019.1645151 31498006

[86] Wen S Zeng M Li Y Hu X Li S Liang X Downregulation of MANCR inhibits cancer cell proliferation in mantle cell lymphoma possibly by interacting with RUNX2. Acta Biochim Biophys Sin (Shanghai) (2019) 51(11):1142–7. 10.1093/abbs/gmz114 31650163

[87] Hu G Gupta SK Troska TP Nair A Gupta M Long non-coding RNA profile in mantle cell lymphoma identifies a functional lncRNA ROR1-AS1 associated with EZH2/PRC2 complex. Oncotarget (2017) 8(46):80223–34. 10.18632/oncotarget.17956 PMC5655192 29113297

[88] Mei M Wang Y Wang Q Liu Y Song W Zhang M CircCDYL Serves as a New Biomarker in Mantle Cell Lymphoma and Promotes Cell Proliferation. Cancer Manag Res (2019) 11:10215–21. 10.2147/CMAR.S232075 PMC6899078 31819653

[89] Handal B Enlow R Lara D Bailey M Vega F Hu P Investigating the Expression of Oncogenic and Tumor Suppressive MicroRNA in DLBCL. J Assoc Genet Technol (2013) 39(1):14–20. 26030163

[90] Lawrie CH Soneji S Marafioti T Cooper CD Palazzo S Paterson JC MicroRNA expression distinguishes between germinal center B cell-like and activated B cell-like subtypes of diffuse large B cell lymphoma. Int J Cancer (2007) 121(5):1156–61. 10.1002/ijc.22800 17487835

[91] Song J Shao Q Li C Liu H Li J Wang Y Effects of microRNA-21 on apoptosis by regulating the expression of PTEN in diffuse large B-cell lymphoma. Med (Baltimore) (2017) 96(39):e7952. 10.1097/MD.0000000000007952 PMC5626260 28953617

[92] Liu K Du J Ruan L MicroRNA-21 regulates the viability and apoptosis of diffuse large B-cell lymphoma cells by upregulating B cell lymphoma-2. Exp Ther Med (2017) 14(5):4489–96. 10.3892/etm.2017.5021 PMC5647720 29067124

[93] Go H Jang JY Kim PJ Kim YG Nam SJ Paik JH MicroRNA-21 plays an oncogenic role by targeting FOXO1 and activating the PI3K/AKT pathway in diffuse large B-cell lymphoma. Oncotarget (2015) 6(17):15035–49. 10.18632/oncotarget.3729 PMC4558134 25909227

[94] Testa U Pelosi E Castelli G Labbaye C miR-146 and miR-155: Two Key Modulators of Immune Response and Tumor Development. Noncoding RNA (2017) 3(3):22. 10.3390/ncrna3030022 PMC5831915 29657293

[95] Pedersen IM Otero D Kao E Miletic AV Hother C Ralfkiaer E Onco-miR-155 targets SHIP1 to promote TNFalpha-dependent growth of B cell lymphomas. EMBO Mol Med (2009) 1(5):288–95. 10.1002/emmm.200900028 PMC2771872 19890474

[96] Zhong H Xu L Zhong JH Xiao F Liu Q Huang HH Clinical and prognostic significance of miR-155 and miR-146a expression levels in formalin-fixed/paraffin-embedded tissue of patients with diffuse large B-cell lymphoma. Exp Ther Med (2012) 3(5):763–70. 10.3892/etm.2012.502 PMC3438582 22969965

[97] Marques SC Ranjbar B Laursen MB Falgreen S Bilgrau AE Bødker JS High miR-34a expression improves response to doxorubicin in diffuse large B-cell lymphoma. Exp Hematol (2016) 44(4):238–46.e2. 10.1016/j.exphem.2015.12.007 26854484

[98] Jia YJ Liu ZB Wang WG Sun CB Wei P Yang YL HDAC6 regulates microRNA-27b that suppresses proliferation, promotes apoptosis and target MET in diffuse large B-cell lymphoma. Leukemia (2018) 32(3):703–11. 10.1038/leu.2017.299 29135973

[99] Verma A Jiang Y Du W Fairchild L Melnick A Elemento O Transcriptome sequencing reveals thousands of novel long non-coding RNAs in B cell lymphoma. Genome Med (2015) 7:110. 10.1186/s13073-015-0230-7 26521025 PMC4628784

[100] Savage KJ Monti S Kutok JL Cattoretti G Neuberg D De Leval L The molecular signature of mediastinal large B-cell lymphoma differs from that of other diffuse large B-cell lymphomas and shares features with classical Hodgkin lymphoma. Blood (2003) 102(12):3871–9. 10.1182/blood-2003-06-1841 12933571

[101] Eis PS Tam W Sun L Chadburn A Li Z Gomez MF Accumulation of miR-155 and BIC RNA in human B cell lymphomas. Proc Natl Acad Sci USA (2005) 102(10):3627–32. 10.1073/pnas.0500613102 PMC552785 15738415

[102] Lim EL Trinh DL Scott DW Chu A Krzywinski M Zhao Y Comprehensive miRNA sequence analysis reveals survival differences in diffuse large B-cell lymphoma patients. Genome Biol (2015) 16(1):18. 10.1186/s13059-014-0568-y 25723320 PMC4308918

[103] Leucci E Cocco M Onnis A De Falco G van Cleef P Bellan C MYC translocation-negative classical Burkitt lymphoma cases: an alternative pathogenetic mechanism involving miRNA deregulation. J Pathol (2008) 216(4):440–50. 10.1002/path.2410 18802929

[104] Bueno MJ Gómez de Cedrón M Gómez-López G Pérez de Castro I Di Lisio L Montes-Moreno S Combinatorial effects of microRNAs to suppress the Myc oncogenic pathway. Blood (2011) 117(23):6255–66. 10.1182/blood-2010-10-315432 21478429

[105] Sampson VB Rong NH Han J Yang Q Aris V Soteropoulos P MicroRNA let-7a down-regulates MYC and reverts MYC-induced growth in Burkitt lymphoma cells. Cancer Res (2007) 67(20):9762–70. 10.1158/0008-5472.CAN-07-2462 17942906

[106] Hummel M Bentink S Berger H Klapper W Wessendorf S Barth TF A biologic definition of Burkitt’s lymphoma from transcriptional and genomic profiling. N Engl J Med (2006) 354(23):2419–30. 10.1056/NEJMoa055351 16760442

[107] Lenze D Leoncini L Hummel M Volinia S Liu CG Amato T The different epidemiologic subtypes of Burkitt lymphoma share a homogenous micro RNA profile distinct from diffuse large B-cell lymphoma. Leukemia (2011) 25(12):1869–76. 10.1038/leu.2011.156 PMC3902789 21701491

[108] Haferlach C Dicker F Schnittger S Kern W Haferlach T Comprehensive genetic characterization of CLL: a study on 506 cases analysed with chromosome banding analysis, interphase FISH, IgV(H) status and immunophenotyping. Leukemia (2007) 21(12):2442–51. 10.1038/sj.leu.2404935 17805327

[109] Cimmino A Calin GA Fabbri M Iorio MV Ferracin M Shimizu M miR-15 and miR-16 induce apoptosis by targeting BCL2. Proc Natl Acad Sci USA (2005) 102(39):13944–9. 10.1073/pnas.0506654102 PMC1236577 16166262

[110] Klein U Lia M Crespo M Siegel R Shen Q Mo T The DLEU2/miR-15a/16-1 cluster controls B cell proliferation and its deletion leads to chronic lymphocytic leukemia. Cancer Cell (2010) 17(1):28–40. 10.1016/j.ccr.2009.11.019 20060366

[111] Visone R Veronese A Rassenti LZ Balatti V Pearl DK Acunzo M miR-181b is a biomarker of disease progression in chronic lymphocytic leukemia. Blood (2011) 118(11):3072–9. 10.1182/blood-2011-01-333484 PMC3175784 21636858

[112] Santanam U Zanesi N Efanov A Costinean S Palamarchuk A Hagan JP Chronic lymphocytic leukemia modeled in mouse by targeted miR-29 expression. Proc Natl Acad Sci USA (2010) 107(27):12210–5. 10.1073/pnas.1007186107 PMC2901490 20566844

[113] Zauli G Voltan R di Iasio MG Bosco R Melloni E Sana ME miR-34a induces the downregulation of both E2F1 and B-Myb oncogenes in leukemic cells. Clin Cancer Res (2011) 17(9):2712–24. 10.1158/1078-0432.CCR-10-3244 21367750

[114] Vargova K Curik N Burda P Basova P Kulvait V Pospisil V MYB transcriptionally regulates the miR-155 host gene in chronic lymphocytic leukemia. Blood (2011) 117(14):3816–25. 10.1182/blood-2010-05-285064 21296997

[115] Frenquelli M Muzio M Scielzo C Fazi C Scarfò L Rossi C MicroRNA and proliferation control in chronic lymphocytic leukemia: functional relationship between miR-221/222 cluster and p27. Blood (2010) 115(19):3949–59. 10.1182/blood-2009-11-254656 20203269

[116] Pekarsky Y Santanam U Cimmino A Palamarchuk A Efanov A Maximov V Tcl1 expression in chronic lymphocytic leukemia is regulated by miR-29 and miR-181. Cancer Res (2006) 66(24):11590–3. 10.1158/0008-5472.CAN-06-3613 17178851

[117] Navarro A Gaya A Martinez A Urbano-Ispizua A Pons A Balagué O MicroRNA expression profiling in classic Hodgkin lymphoma. Blood (2008) 111(5):2825–32. 10.1182/blood-2007-06-096784 18089852

[118] Zhu DX Zhu W Fang C Fan L Zou ZJ Wang YH miR-181a/b significantly enhances drug sensitivity in chronic lymphocytic leukemia cells via targeting multiple anti-apoptosis genes. Carcinogenesis (2012) 33(7):1294–301. 10.1093/carcin/bgs179 22610076

[119] Calin GA Ferracin M Cimmino A Di Leva G Shimizu M Wojcik SE A MicroRNA signature associated with prognosis and progression in chronic lymphocytic leukemia. N Engl J Med (2005) 353(17):1793–801. 10.1056/NEJMoa050995 16251535

[120] Sattari A Siddiqui H Moshiri F Ngankeu A Nakamura T Kipps TJ Upregulation of long noncoding RNA MIAT in aggressive form of chronic lymphocytic leukemias. Oncotarget (2016) 7(34):54174–82. 10.18632/oncotarget.11099 PMC5338916 27527866

[121] Pan Y Li H Guo Y Luo Y Li H Xu Y A pilot study of long noncoding RNA expression profiling by microarray in follicular lymphoma. Gene (2016) 577(2):132–9. 10.1016/j.gene.2015.11.029 26692150

[122] Dahl M Daugaard I Andersen MS Hansen TB Grønbæk K Kjems J Enzyme-free digital counting of endogenous circular RNA molecules in B-cell malignancies. Lab Invest (2018) 98(12):1657–69. 10.1038/s41374-018-0108-6 PMC6265260 30087459

[123] Li C Kim SW Rai D Bolla AR Adhvaryu S Kinney MC Copy number abnormalities, MYC activity, and the genetic fingerprint of normal B cells mechanistically define the microRNA profile of diffuse large B-cell lymphoma. Blood (2009) 113(26):6681–90. 10.1182/blood-2009-01-202028 PMC3401058 19278952

[124] Visone R Rassenti LZ Veronese A Taccioli C Costinean S Aguda BD Karyotype-specific microRNA signature in chronic lymphocytic leukemia. Blood (2009) 114(18):3872–9. 10.1182/blood-2009-06-229211 PMC2773482 19717645

[125] Lenz G Wright GW Emre NC Kohlhammer H Dave SS Davis RE Molecular subtypes of diffuse large B-cell lymphoma arise by distinct genetic pathways. Proc Natl Acad Sci U S A (2008) 105: (36):13520–5. 10.1073/pnas.0804295105 PMC2533222 18765795

[126] Scholtysik R Kreuz M Klapper W Burkhardt B Feller AC Hummel M Detection of genomic aberrations in molecularly defined Burkitt’s lymphoma by array-based, high resolution, single nucleotide polymorphism analysis. Haematologica (2010) 95(12):2047–55. 10.3324/haematol.2010.026831 PMC2995562 20823134

[127] Chapiro E Russell LJ Struski S Cavé H Radford-Weiss I Valle VD A new recurrent translocation t(11;14)(q24;q32) involving IGH@ and miR-125b-1 in B-cell progenitor acute lymphoblastic leukemia. Leukemia (2010) 24(7):1362–4. 10.1038/leu.2010.93 20485370

[128] Kminkova J Mraz M Zaprazna K Navrkalova V Tichy B Plevova K Identification of novel sequence variations in microRNAs in chronic lymphocytic leukemia. Carcinogenesis (2014) 35(5):992–1002. 10.1093/carcin/bgt396 24306027 PMC4004199

[129] Kwanhian W Lenze D Alles J Motsch N Barth S Döll C MicroRNA-142 is mutated in about 20% of diffuse large B-cell lymphoma. Cancer Med (2012) 1(2):141–55. 10.1002/cam4.29 PMC3544448 23342264

[130] Pallasch CP Patz M Park YJ Hagist S Eggle D Claus R miRNA deregulation by epigenetic silencing disrupts suppression of the oncogene PLAG1 in chronic lymphocytic leukemia. Blood (2009) 114(15):3255–64. 10.1182/blood-2009-06-229898 PMC2925729 19692702

[131] Chim CS Wong KY Leung CY Chung LP Hui PK Chan SY Epigenetic inactivation of the hsa-miR-203 in haematological malignancies. J Cell Mol Med (2011) 15(12):2760–7. 10.1111/j.1582-4934.2011.01274.x PMC4373446 21323860

[132] Wong KY So CC Loong F Chung LP Lam WW Liang R Epigenetic inactivation of the miR-124-1 in haematological malignancies. PloS One (2011) 6(4):e19027. 10.1371/journal.pone.0019027 21544199 PMC3081325

[133] Sampath D Liu C Vasan K Sulda M Puduvalli VK Wierda WG Histone deacetylases mediate the silencing of miR-15a, miR-16, and miR-29b in chronic lymphocytic leukemia. Blood (2012) 119(5):1162–72. 10.1182/blood-2011-05-351510 PMC3277352 22096249

[134] Zhang X Zhao X Fiskus W Lin J Lwin T Rao R Coordinated silencing of MYC-mediated miR-29 by HDAC3 and EZH2 as a therapeutic target of histone modification in aggressive B-Cell lymphomas. Cancer Cell (2012) 22(4):506–23. 10.1016/j.ccr.2012.09.003 PMC3973134 23079660

[135] Zhang X Chen X Lin J Lwin T Wright G Moscinski LC Myc represses miR-15a/miR-16-1 expression through recruitment of HDAC3 in mantle cell and other non-Hodgkin B-cell lymphomas. Oncogene (2012) 31(24):3002–8. 10.1038/onc.2011.470 PMC3982396 22002311

[136] Mihailovich M Bremang M Spadotto V Musiani D Vitale E Varano G miR-17-92 fine-tunes MYC expression and function to ensure optimal B cell lymphoma growth. Nat Commun (2015) 6:8725. 10.1038/ncomms9725 26555894 PMC4667639

[137] Sander S Bullinger L Klapproth K Fiedler K Kestler HA Barth TF MYC stimulates EZH2 expression by repression of its negative regulator miR-26a. Blood (2008) 112(10):4202–12. 10.1182/blood-2008-03-147645 18713946

[138] Sotillo E Laver T Mellert H Schelter JM Cleary MA McMahon S Myc overexpression brings out unexpected antiapoptotic effects of miR-34a. Oncogene (2011) 30(22):2587–94. 10.1038/onc.2010.634 PMC3128883 21297663

[139] Schneider C Setty M Holmes AB Maute RL Leslie CS Mussolin L MicroRNA 28 controls cell proliferation and is down-regulated in B-cell lymphomas. Proc Natl Acad Sci USA (2014) 111(22):8185–90. 10.1073/pnas.1322466111 PMC4050621 24843176

[140] Mraz M Malinova K Kotaskova J Pavlova S Tichy B Malcikova J miR-34a, miR-29c and miR-17-5p are downregulated in CLL patients with TP53 abnormalities. Leukemia (2009) 23(6):1159–63. 10.1038/leu.2008.377 19158830

[141] Cerna K Oppelt J Chochola V Musilova K Seda V Pavlasova G MicroRNA miR-34a downregulates FOXP1 during DNA damage response to limit BCR signalling in chronic lymphocytic leukaemia B cells. Leukemia (2019) 33(2):403–14. 10.1038/s41375-018-0230-x 30111844

[142] Fabbri M Bottoni A Shimizu M Spizzo R Nicoloso MS Rossi S Association of a microRNA/TP53 feedback circuitry with pathogenesis and outcome of B-cell chronic lymphocytic leukemia. Jama (2011) 305(1):59–67. 10.1001/jama.2010.1919 21205967 PMC3690301

[143] Jeong D Kim J Nam J Sun H Lee YH Lee TJ MicroRNA-124 links p53 to the NF-κB pathway in B-cell lymphomas. Leukemia (2015) 29(9):1868–74. 10.1038/leu.2015.101 25915824

[144] Blume CJ Hotz-Wagenblatt A Hüllein J Sellner L Jethwa A Stolz T p53-dependent non-coding RNA networks in chronic lymphocytic leukemia. Leukemia (2015) 29(10):2015–23. 10.1038/leu.2015.119 25971364

[145] Gatto G Rossi A Rossi D Kroening S Bonatti S Mallardo M Epstein-Barr virus latent membrane protein 1 trans-activates miR-155 transcription through the NF-kappaB pathway. Nucleic Acids Res (2008) 36(20):6608–19. 10.1093/nar/gkn666 PMC2582607 18940871

[146] Bai H Wei J Deng C Yang X Wang C Xu R MicroRNA-21 regulates the sensitivity of diffuse large B-cell lymphoma cells to the CHOP chemotherapy regimen. Int J Hematol (2013) 97(2):223–31. 10.1007/s12185-012-1256-x 23275230

[147] Allegra D Bilan V Garding A Döhner H Stilgenbauer S Kuchenbauer F Defective DROSHA processing contributes to downregulation of MiR-15/-16 in chronic lymphocytic leukemia. Leukemia (2014) 28(1):98–107. 10.1038/leu.2013.246 23974981

[148] Anastasiadou E Boccellato F Vincenti S Rosato P Bozzoni I Frati L Epstein-Barr virus encoded LMP1 downregulates TCL1 oncogene through miR-29b. Oncogene (2010) 29(9):1316–28. 10.1038/onc.2009.439 19966860

[149] Cameron JE Yin Q Fewell C Lacey M McBride J Wang X Epstein-Barr virus latent membrane protein 1 induces cellular MicroRNA miR-146a, a modulator of lymphocyte signaling pathways. J Virol (2008) 82(4):1946–58. 10.1128/JVI.02136-07 PMC2258704 18057241

[150] Rosato P Anastasiadou E Garg N Lenze D Boccellato F Vincenti S Differential regulation of miR-21 and miR-146a by Epstein-Barr virus-encoded EBNA2. Leukemia (2012) 26(11):2343–52. 10.1038/leu.2012.108 PMC3496086 22614176

[151] Anastasiadou E Stroopinsky D Alimperti S Jiao AL Pyzer AR Cippitelli C Epstein-Barr virus-encoded EBNA2 alters immune checkpoint PD-L1 expression by downregulating miR-34a in B-cell lymphomas. Leukemia (2019) 33(1):132–47. 10.1038/s41375-018-0178-x PMC6327052 29946193

[152] Wood CD Carvell T Gunnell A Ojeniyi OO Osborne C West MJ Enhancer Control of MicroRNA miR-155 Expression in Epstein-Barr Virus-Infected B Cells. J Virol (2018) 92(19):e00716-18. 10.1128/JVI.00716-18 30021904 PMC6146817

[153] Ayoubian H Ludwig N Fehlmann T Menegatti J Gröger L Anastasiadou E Epstein-Barr Virus Infection of Cell Lines Derived from Diffuse Large B-Cell Lymphomas Alters MicroRNA Loading of the Ago2 Complex. J Virol (2019) 93(3):e01297-18. 10.1128/JVI.01297-18 30429351 PMC6340033

[154] Marquitz AR Mathur A Edwards RH Raab-Traub N Host Gene Expression Is Regulated by Two Types of Noncoding RNAs Transcribed from the Epstein-Barr Virus BamHI A Rightward Transcript Region. J Virol (2015) 89(22):11256–68. 10.1128/JVI.01492-15 PMC4645673 26311882

[155] Bernhardt K Haar J Tsai M-H Poirey R Feederle R Delecluse H-J A Viral microRNA Cluster Regulates the Expression of PTEN, p27 and of a bcl-2 Homolog. PloS Pathog (2016) 12(1):e1005405. 10.1371/journal.ppat.1005405 26800049 PMC4723338

[156] Židovec Lepej S Matulić M Gršković P Pavlica M Radmanić L Korać P miRNAs: EBV Mechanism for Escaping Host’s Immune Response and Supporting Tumorigenesis. Pathogens (2020) 9(5):353. 10.3390/pathogens9050353 PMC7281681 32397085

[157] Murer A Rühl J Zbinden A Capaul R Hammerschmidt W Chijioke O MicroRNAs of Epstein-Barr Virus Attenuate T-Cell-Mediated Immune Control In Vivo. mBio (2019) 10(1):e01941-18. 10.1128/mBio.01941-18 PMC6336420 30647153

[158] Zhang J Li X Hu J Cao P Yan Q Zhang S Long noncoding RNAs involvement in Epstein-Barr virus infection and tumorigenesis. Virol J (2020) 17(1):51. 10.1186/s12985-020-01308-y 32272952 PMC7146903

[159] Huang JT Chen JN Gong LP Bi YH Liang J Zhou L Identification of virus-encoded circular RNA. Virology (2019) 529:144–51. 10.1016/j.virol.2019.01.014 30710798

[160] Gottwein E Corcoran DL Mukherjee N Skalsky RL Hafner M Nusbaum JD Viral MicroRNA Targetome of KSHV-Infected Primary Effusion Lymphoma Cell Lines. Cell Host Microbe (2011) 10(5):515–26. 10.1016/j.chom.2011.09.012 PMC3222872 22100165

[161] Tagawa T Gao S Koparde VN Gonzalez M Spouge JL Serquiña AP Discovery of Kaposi’s sarcoma herpesvirus-encoded circular RNAs and a human antiviral circular RNA. Proc Natl Acad Sci U S A (2018) 115: (50):12805–10. 10.1073/pnas.1816183115 PMC6294913 30455306

[162] Liu J Chen G Feng L Zhang W Pelicano H Wang F Loss of p53 and altered miR15-a/16-1MCL-1 pathway in CLL: insights from TCL1-Tg:p53(-/-) mouse model and primary human leukemia cells. Leukemia (2014) 28(1):118–28. 10.1038/leu.2013.125 PMC3806892 23608884

[163] Rossi S Shimizu M Barbarotto E Nicoloso MS Dimitri F Sampath D microRNA fingerprinting of CLL patients with chromosome 17p deletion identify a miR-21 score that stratifies early survival. Blood (2010) 116(6):945–52. 10.1182/blood-2010-01-263889 PMC4916575 20393129

[164] Navarro A Diaz T Martinez A Gaya A Pons A Gel B Regulation of JAK2 by miR-135a: prognostic impact in classic Hodgkin lymphoma. Blood (2009) 114(14):2945–51. 10.1182/blood-2009-02-204842 19666866

[165] Yamakuchi M Ferlito M Lowenstein CJ miR-34a repression of SIRT1 regulates apoptosis. Proc Natl Acad Sci USA (2008) 105(36):13421–6. 10.1073/pnas.0801613105 PMC2533205 18755897

[166] Chang TC Wentzel EA Kent OA Ramachandran K Mullendore M Lee KH Transactivation of miR-34a by p53 broadly influences gene expression and promotes apoptosis. Mol Cell (2007) 26(5):745–52. 10.1016/j.molcel.2007.05.010 PMC1939978 17540599

[167] Sampath D Calin GA Puduvalli VK Gopisetty G Taccioli C Liu CG Specific activation of microRNA106b enables the p73 apoptotic response in chronic lymphocytic leukemia by targeting the ubiquitin ligase Itch for degradation. Blood (2009) 113(16):3744–53. 10.1182/blood-2008-09-178707 PMC2670791 19096009

[168] Olive V Bennett MJ Walker JC Ma C Jiang I Cordon-Cardo C miR-19 is a key oncogenic component of mir-17-92. Genes Dev (2009) 23(24):2839–49. 10.1101/gad.1861409 PMC2800084 20008935

[169] Yamanaka Y Tagawa H Takahashi N Watanabe A Guo YM Iwamoto K Aberrant overexpression of microRNAs activate AKT signaling via down-regulation of tumor suppressors in natural killer-cell lymphoma/leukemia. Blood (2009) 114(15):3265–75. 10.1182/blood-2009-06-222794 19641183

[170] Palacios F Abreu C Prieto D Morande P Ruiz S Fernández-Calero T Activation of the PI3K/AKT pathway by microRNA-22 results in CLL B-cell proliferation. Leukemia (2015) 29(1):115–25. 10.1038/leu.2014.158 24825182

[171] Cui B Chen L Zhang S Mraz M Fecteau JF Yu J MicroRNA-155 influences B-cell receptor signaling and associates with aggressive disease in chronic lymphocytic leukemia. Blood (2014) 124(4):546–54. 10.1182/blood-2014-03-559690 PMC4110661 24914134

[172] Mraz M Kipps TJ MicroRNAs and B cell receptor signaling in chronic lymphocytic leukemia. Leuk Lymphoma (2013) 54(8):1836–9. 10.3109/10428194.2013.796055 PMC4144718 23597135

[173] Kim JH Kim WS Park C Epstein-Barr virus latent membrane protein-1 protects B-cell lymphoma from rituximab-induced apoptosis through miR-155-mediated Akt activation and up-regulation of Mcl-1. Leuk Lymphoma (2012) 53(8):1586–91. 10.3109/10428194.2012.659736 22268450

[174] Boysen J Sinha S Price-Troska T Warner SL Bearss DJ Viswanatha D The tumor suppressor axis p53/miR-34a regulates Axl expression in B-cell chronic lymphocytic leukemia: implications for therapy in p53-defective CLL patients. Leukemia (2014) 28(2):451–5. 10.1038/leu.2013.298 PMC3929965 24217154

[175] Pekarsky Y Koval A Hallas C Bichi R Tresini M Malstrom S Tcl1 enhances Akt kinase activity and mediates its nuclear translocation. Proc Natl Acad Sci USA (2000) 97(7):3028–33. 10.1073/pnas.97.7.3028 PMC16186 10716693

[176] Zhao X Tian X Knockdown of long noncoding RNA HOTAIR inhibits cell growth of human lymphoma cells by upregulation of miR-148b. J Cell Biochem (2019) 120(8):12348–59. 10.1002/jcb.28500 30848513

[177] Shim H Nam J Kim SW NF-κB p65 represses microRNA-124 transcription in diffuse large B-cell lymphoma. Genes Genomics (2020) 42(5):543–51. 10.1007/s13258-020-00922-y 32207045

[178] Chen RW Bemis LT Amato CM Myint H Tran H Birks DK Truncation in CCND1 mRNA alters miR-16-1 regulation in mantle cell lymphoma. Blood (2008) 112(3):822–9. 10.1182/blood-2008-03-142182 PMC2481543 18483394

[179] O’Donnell KA Wentzel EA Zeller KI Dang CV Mendell JT c-Myc-regulated microRNAs modulate E2F1 expression. Nature (2005) 435(7043):839–43. 10.1038/nature03677 15944709

[180] Li LJ Chai Y Guo XJ Chu SL Zhang LS The effects of the long non-coding RNA MALAT-1 regulated autophagy-related signaling pathway on chemotherapy resistance in diffuse large B-cell lymphoma. BioMed Pharmacother (2017) 89:939–48. 10.1016/j.biopha.2017.02.011 28292022

[181] Pickard MR Williams GT Molecular and Cellular Mechanisms of Action of Tumour Suppressor GAS5 LncRNA. Genes (Basel) (2015) 6(3):484–99. 10.3390/genes6030484 PMC4584312 26198250

[182] Hu G Lou Z Gupta M The long non-coding RNA GAS5 cooperates with the eukaryotic translation initiation factor 4E to regulate c-Myc translation. PloS One (2014) 9(9):e107016. 10.1371/journal.pone.0107016 25197831 PMC4157848

[183] Ding L Zhang Y Han L Fu L Mei X Wang J Activating and sustaining c-Myc by depletion of miR-144/451 gene locus contributes to B-lymphomagenesis. Oncogene (2018) 37(10):1293–307. 10.1038/s41388-017-0055-5 PMC6168470 29284789

[184] Mu P Han YC Betel D Yao E Squatrito M Ogrodowski P Genetic dissection of the miR-17~92 cluster of microRNAs in Myc-induced B-cell lymphomas. Genes Dev (2009) 23(24):2806–11. 10.1101/gad.1872909 PMC2800095 20008931

[185] Olive V Sabio E Bennett MJ De Jong CS Biton A McGann JC A component of the mir-17-92 polycistronic oncomir promotes oncogene-dependent apoptosis. Elife (2013) 2:e00822. 10.7554/eLife.00822 24137534 PMC3796314

[186] Medina PP Nolde M Slack FJ OncomiR addiction in an in vivo model of microRNA-21-induced pre-B-cell lymphoma. Nature (2010) 467(7311):86–90. 10.1038/nature09284 20693987

[187] Lin J Lwin T Zhao JJ Tam W Choi YS Moscinski LC Follicular dendritic cell-induced microRNA-mediated upregulation of PRDM1 and downregulation of BCL-6 in non-Hodgkin’s B-cell lymphomas. Leukemia (2011) 25(1):145–52. 10.1038/leu.2010.230 PMC3083119 20966935

[188] Lwin T Zhao X Cheng F Zhang X Huang A Shah B A microenvironment-mediated c-Myc/miR-548m/HDAC6 amplification loop in non-Hodgkin B cell lymphomas. J Clin Invest (2013) 123(11):4612–26. 10.1172/JCI64210 PMC3809771 24216476

[189] Craig VJ Cogliatti SB Imig J Renner C Neuenschwander S Rehrauer H Myc-mediated repression of microRNA-34a promotes high-grade transformation of B-cell lymphoma by dysregulation of FoxP1. Blood (2011) 117(23):6227–36. 10.1182/blood-2010-10-312231 PMC3790944 21460242

[190] Psathas JN Doonan PJ Raman P Freedman BD Minn AJ Thomas-Tikhonenko A The Myc-miR-17-92 axis amplifies B-cell receptor signaling via inhibition of ITIM proteins: a novel lymphomagenic feed-forward loop. Blood (2013) 122(26):4220–9. 10.1182/blood-2012-12-473090 PMC3868926 24169826

[191] Jablonska E Gorniak P Szydlowski M Sewastianik T Bialopiotrowicz E Polak A MiR-17-92 represses PTPROt and PP2A phosphatases and amplifies tonic BCR signaling in DLBCL cells. Exp Hematol (2017) 46:56–61.e1. 10.1016/j.exphem.2016.09.011 27720936

[192] Chen L Widhopf G Huynh L Rassenti L Rai KR Weiss A Expression of ZAP-70 is associated with increased B-cell receptor signaling in chronic lymphocytic leukemia. Blood (2002) 100(13):4609–14. 10.1182/blood-2002-06-1683 12393534

[193] Mraz M Dolezalova D Plevova K Stano Kozubik K Mayerova V Cerna K MicroRNA-650 expression is influenced by immunoglobulin gene rearrangement and affects the biology of chronic lymphocytic leukemia. Blood (2012) 119(9):2110–3. 10.1182/blood-2011-11-394874 22234685

[194] Mraz M Stano Kozubik K Plevova K Musilova K Tichy B Borsky M The origin of deletion 22q11 in chronic lymphocytic leukemia is related to the rearrangement of immunoglobulin lambda light chain locus. Leuk Res (2013) 37(7):802–8. 10.1016/j.leukres.2013.03.018 23608880

[195] Yin Q Wang X McBride J Fewell C Flemington E B-cell receptor activation induces BIC/miR-155 expression through a conserved AP-1 element. J Biol Chem (2008) 283(5):2654–62. 10.1074/jbc.M708218200 PMC2810639 18048365

[196] Zheng Z Sun R Zhao HJ Fu D Zhong HJ Weng XQ MiR155 sensitized B-lymphoma cells to anti-PD-L1 antibody via PD-1/PD-L1-mediated lymphoma cell interaction with CD8+T cells. Mol Cancer (2019) 18(1):54. 10.1186/s12943-019-0977-3 30925928 PMC6441197

[197] Poles WA Nishi EE de Oliveira MB Eugênio AIP de Andrade TA Campos AHFM Targeting the polarization of tumor-associated macrophages and modulating mir-155 expression might be a new approach to treat diffuse large B-cell lymphoma of the elderly. Cancer Immunol Immunother (2019) 68(2):269–82. 10.1007/s00262-018-2273-2 PMC11028330 30430204

[198] Zonari E Pucci F Saini M Mazzieri R Politi LS Gentner B A role for miR-155 in enabling tumor-infiltrating innate immune cells to mount effective antitumor responses in mice. Blood (2013) 122(2):243–52. 10.1182/blood-2012-08-449306 23487026

[199] Lennox KA Behlke MA Chemical modification and design of anti-miRNA oligonucleotides. Gene Ther (2011) 18(12):1111–20. 10.1038/gt.2011.100 21753793

[200] Zhang Y Roccaro AM Rombaoa C Flores L Obad S Fernandes SM LNA-mediated anti-miR-155 silencing in low-grade B-cell lymphomas. Blood (2012) 120(8):1678–86. 10.1182/blood-2012-02-410647 22797699

[201] Seto AG Beatty X Lynch JM Hermreck M Tetzlaff M Duvic M Cobomarsen, an oligonucleotide inhibitor of miR-155, co-ordinately regulates multiple survival pathways to reduce cellular proliferation and survival in cutaneous T-cell lymphoma. Br J Haematol (2018) 183(3):428–44. 10.1111/bjh.15547 30125933

[202] Amodio N Stamato MA Juli G Morelli E Fulciniti M Manzoni M Drugging the lncRNA MALAT1 via LNA gapmeR ASO inhibits gene expression of proteasome subunits and triggers anti-multiple myeloma activity. Leukemia (2018) 32(9):1948–57. 10.1038/s41375-018-0067-3 PMC6127082 29487387

[203] Zhou CC Yang F Yuan SX Ma JZ Liu F Yuan JH Systemic genome screening identifies the outcome associated focal loss of long noncoding RNA PRAL in hepatocellular carcinoma. Hepatology (2016) 63(3):850–63. 10.1002/hep.28393 26663434

[204] Liu N Dai Q Zheng G He C Parisien M Pan T N(6)-methyladenosine-dependent RNA structural switches regulate RNA-protein interactions. Nature (2015) 518(7540):560–4. 10.1038/nature14234 PMC4355918 25719671

[205] Zuo X Chen Z Gao W Zhang Y Wang J Wang J M6A-mediated upregulation of LINC00958 increases lipogenesis and acts as a nanotherapeutic target in hepatocellular carcinoma. J Hematol Oncol (2020) 13(1):5. 10.1186/s13045-019-0839-x 31915027 PMC6951025

[206] Beheshti A Stevenson K Vanderburg C Ravi D McDonald JT Christie AL Identification of Circulating Serum Multi-MicroRNA Signatures in Human DLBCL Models. Sci Rep (2019) 9(1):17161. 10.1038/s41598-019-52985-x 31748664 PMC6868195

[207] Tang JL Li XM Zhang L [Expression and Significance of LncRNA RP11-513G11.1 in Peripheral Blood of Patients with Diffuse Large B-Cell Lymphoma]. Zhongguo Shi Yan Xue Ye Xue Za Zhi (2019) 27(5):1515–21. 10.19746/j.cnki.issn.1009-2137.2019.05.024 31607306

[208] Zhang X Xie K Zhou H Wu Y Li C Liu Y Role of non-coding RNAs and RNA modifiers in cancer therapy resistance. Mol Cancer (2020) 19(1):47. 10.1186/s12943-020-01171-z 32122355 PMC7050132

[209] Huang Q Xia J Wang L Wang X Ma X Deng Q miR-153 suppresses IDO1 expression and enhances CAR T cell immunotherapy. J Hematol Oncol (2018) 11(1):58. 10.1186/s13045-018-0600-x 29685162 PMC5914051

[210] Lin R Sampson JH Li QJ Zhu B miR-23a blockade enhances adoptive T cell transfer therapy by preserving immune-competence in the tumor microenvironment. Oncoimmunology (2015) 4(3):e990803. 10.4161/2162402X.2014.990803 25949909 PMC4404905

